# Phenome-wide analysis of genome-wide polygenic scores

**DOI:** 10.1038/mp.2015.126

**Published:** 2015-08-25

**Authors:** E Krapohl, J Euesden, D Zabaneh, J-B Pingault, K Rimfeld, S von Stumm, P S Dale, G Breen, P F O'Reilly, R Plomin

**Affiliations:** 1 MRC Social, Genetic and Developmental Psychiatry Centre, Institute of Psychiatry, Psychology and Neuroscience, King's College London, London, UK; 2 Division of Psychology and Language Sciences, University College London, London, UK; 3 Department of Psychology, Goldsmiths University of London, New Cross, London, UK; 4 Department of Speech and Hearing Sciences, University of New Mexico, Albuquerque, NM, USA

## Abstract

Genome-wide polygenic scores (GPS), which aggregate the effects of thousands of DNA variants from genome-wide association studies (GWAS), have the potential to make genetic predictions for individuals. We conducted a systematic investigation of associations between GPS and many behavioral traits, the behavioral phenome. For 3152 unrelated 16-year-old individuals representative of the United Kingdom, we created 13 GPS from the largest GWAS for psychiatric disorders (for example, schizophrenia, depression and dementia) and cognitive traits (for example, intelligence, educational attainment and intracranial volume). The behavioral phenome included 50 traits from the domains of psychopathology, personality, cognitive abilities and educational achievement. We examined phenome-wide profiles of associations for the entire distribution of each GPS and for the extremes of the GPS distributions. The cognitive GPS yielded stronger predictive power than the psychiatric GPS in our UK-representative sample of adolescents. For example, education GPS explained variation in adolescents' behavior problems (~0.6%) and in educational achievement (~2%) but psychiatric GPS were associated with neither. Despite the modest effect sizes of current GPS, quantile analyses illustrate the ability to stratify individuals by GPS and opportunities for research. For example, the highest and lowest septiles for the education GPS yielded a 0.5 s.d. difference in mean math grade and a 0.25 s.d. difference in mean behavior problems. We discuss the usefulness and limitations of GPS based on adult GWAS to predict genetic propensities earlier in development.

## Introduction

One of the most striking findings emerging from genome-wide association studies (GWAS) of complex traits is the scarcity of common single nucleotide polymorphism (SNP) associations that account for more than 1% of trait variation in the population.^1, 2^ Although GWAS have been successful in discovering and replicating SNP associations for many traits and disorders,^3^ the dearth of larger SNP associations in well-powered GWAS demonstrates that the ubiquitous heritability of complex dimensions and common disorders is caused by thousands of common DNA variants of small effect.^1, 4^ Because their effects are miniscule, a single common SNP is of little use for prediction. For this reason, the future of genetic prediction lies with polygenic scores that aggregate the effects of thousands of SNPs discovered by GWAS, including variants that do not achieve genome-wide significance.^5^ Unlike quantitative genetic designs that estimate the net effect of DNA differences in a population—such as twin and adoption studies and SNP-based heritability^6^—polygenic scores provide individual-specific estimates of genetic propensities for specific SNPs.

Here we refer to polygenic scores as genome-wide polygenic scores (GPS) for two reasons. First, the acronym GPS excludes the term ‘risk', in contrast to the previous labels, which imply that genetic influences are inevitably associated with negative outcomes. Second, the acronym GPS in its original use as global positioning system is an apt metaphor for the use of DNA differences across the genome to guide research on genetic influence.

Association statistics for dozens of large meta-analytic GWAS are now available, including GWAS for psychiatric and cognitive traits. The GPS based on these GWAS results are limited by the ‘hidden heritability' ceiling and, as yet, they account for only a few percent of the variance or liability of their target trait.^2^ In addition, most GWAS are based on comparisons between diagnosed cases versus controls using a liability model that assumes continuous liability throughout the population, but the extent to which these case/control results generalize to prediction of continuous traits in the population needs to be established empirically.

Multivariate quantitative genetic analyses using the twin method as well as well as SNP heritability methods have shown that genetic effects are to a substantial extent pleiotropic across complex traits in general^7^ and in particular across cognitive abilities and disabilities^8, 9^ and across psychopathologies.^10, 11, 12, 13^ This pleiotropy suggests the usefulness of going beyond ‘candidate-phenotype' analyses of a single GPS-trait pairing to consider the multivariate profile of GPS associations across many behavioral traits, the behavioral phenome.

Here, we report the first phenome-wide analysis of GPS derived from 13 published major psychiatric, cognitive and biometric GWAS. We applied effect size and significance estimates from GWAS summary statistics to create GPS from raw genotype data for individuals in our target sample. The phenome included 50 traits from the domains of psychopathology, personality, cognitive abilities, and educational achievement, assessed in a representative sample of over 3000 16-year-old individuals in the United Kingdom.

The main focus of this paper is to explore the profile of GPS associations across the behavioral phenome for the entire distribution of each GPS and for the extremes of the GPS distributions. One use of polygenic scores is to predict genetic propensities early in development in order to facilitate interventions that promote potential and prevent problems. As a step in this direction, the present sample consists of adolescents as they finish compulsory schooling at age 16. We test whether GPS, based on current GWAS, predict phenotypic variation in the adolescent population, and we discuss the usefulness and limitations of GPS based on adult GWAS to predict genetic propensities earlier in development.

## Materials and methods

We used genome-wide genotype and phenome-wide behavioral data from 3152 unrelated adolescents drawn from the UK-representative Twins Early Development Study^14, 15, 16^ (Supplementary Table 1). We processed the 3152 genotypes using standard quality control procedures followed by imputation of SNPs using the 1000 Genomes Project reference panel^17^ (Supplementary Methods 1). After quality control, we included around 4.3 million variants into the polygenic score analyses (Supplementary Methods 1). Association analyses were conducted using imputed markers and principal components to control for population stratification. Individuals were assessed on a wide range of phenotypes at the age of 16. The present analyses included 50 traits from the domains of psychopathology, personality, cognitive abilities and educational achievement (Supplementary Methods 2). All measures were age- and sex-regressed and the *z*-scores were used in the analyses.

**Figure 1:**
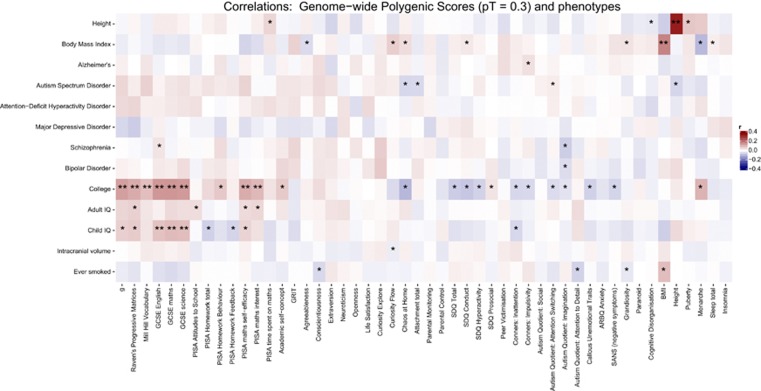
Correlations between 13 genome-wide polygenic scores and 50 traits from the behavioral phenome. These results are based on GPS constructed using a GWAS *P*-value threshold (*P*_T_)=0.30; results for *P*_T_ =0.10 and 0.05 (Supplementary Figures 1a and b and Supplementary Table 3). *P*-values that pass Nyholt–Sidak correction (see Supplementary Methods 1) are indicated with two asterisks, whereas those reaching nominal significance (thus suggestive evidence) are shown with a single asterisk.

We created 13 GPS for each of the over 3000 individuals in our sample using summary statistics from 13 published GWAS^18, 19, 20, 21, 22, 23, 24, 25, 26, 27, 28^ (Supplementary Table 2). Here we present results using a *P*=0.30 threshold for including SNPs from the published GWAS (Figure 1 and Supplementary Table 3); results for GPS based on the *P*-value thresholds of 0.10 and 0.05 are included in the Supplementary material (Supplementary Figures 1a and b and Supplementary Table 3). The selection of the relatively lenient *P*=0.30 threshold was based on the evidence that many associated markers lie within the ensemble of individually non-significant SNPs, with power of the GPS increasing with number of SNP included.^5^ We also report results (Supplementary Figure 2 and Supplementary Table 4) from a high-resolution polygenic scoring approach, implemented in the software PRSice (London, UK), that identifies the most predictive GPS for each phenotype.^29^

We describe two types of main results: (i) associations between GPS and the behavioral phenome for the entire sample, which demonstrate the usefulness of cross-trait prediction, and (ii) quantile analyses showing the association between selected GPS and behavior by septile, which illustrates the ability to stratify individuals by GPS and the potential of polygenic score for phenotype prediction.

To inform these analyses, we demonstrate that GPS are normally distributed and discuss the implications for considering both ends—resilience as well as risk—of GPS distributions. We also examine three types of correlations: (i) genetic correlations between the GWAS summary statistics (ii) correlations between the GPS, and (iii) phenotypic correlations between the target phenotypes. These correlations support the usefulness of a phenome-wide analysis of GPS.

## Results

### GPS are normally distributed

The quantitative genetic model assumes that many genetic variants of small effect drive the heritability of complex traits and common disorders,^30^ even though each marker is inherited in the discrete manner hypothesized by Mendel.^31^ Therefore, the central limit theorem implies that the distribution of polygenic scores in the population will approach normality. Specifically, the normal distribution is to be expected whenever trait variation is polygenic and produced by the addition of a large number of small effects.

Nonetheless, the normality of GPS (Supplementary Figures 3a and c) merits emphasis because it illustrates that common disorders can be considered as extremes of the common polygenic liability spectrum, which has far-reaching implications for diagnosis, treatment and prevention.^32^ It also implies that GPS can be operationalized in terms of ‘resilience' as well as ‘risk' predictors. There is untapped research potential for operationalizing the negative tail of GPS for disorders as ‘resilience' and the negative end of cognitive or education GPS as ‘risk' factors. This ‘other end' of the normal distribution of GPS is uncharted territory. From an evolutionary perspective, averageness might be an adaptive trade-off against the mishmash of costs and benefits of more extreme GPS, especially given the fluctuating nature of selection.^32^

### Intercorrelations between GWAS, GPS and phenotypic traits

As depicted in Supplementary Figure 4 the phenotypic correlations between the target phenotypes in our sample of adolescents show substantial intercorrelations, with a ‘cognitive' and a ‘psychopathology' cluster.

We estimate the genetic correlation between the discovery GWAS using a new technique based on LD score regression,^33, 34^ which uses only GWAS summary statistics and linkage disequilibrium information to decompose true polygenic variance/covariance from confounding (see Supplementary Methods for details). Supplementary Figure 5 depicts the genetic correlations between the 13 GWAS, which provide evidence for significant and substantial pleiotropy. In addition to the genetic correlations reported previously,^34^ we add correlations for the summary statistics of the child IQ GWAS,^19^ adult IQ^35^ and intracranial volume.^20^ The observed genetic correlations replicate and extend previous research. We confirm genetic overlap between the major psychoses^13, 25, 34, 36, 37^ and between cognitive phenotypes including intracranial volume,^18, 20, 38, 39, 40^ respectively. We further find correlations between these two clusters—for example, strong negative associations between the cognitive phenotypes and Alzheimer's and positive associations between educational attainment and autism spectrum disorder as well as bipolar disorder.

We also examined correlations between the GPS created for our sample (Supplementary Figures 6a and c and Supplementary Table 3). We find similar correlation patterns but weaker overall correlations.

These genetic correlations provide evidence that polygenic effects are to a substantial degree pleiotropic across traits. Together with finding substantial correlations between the target phenotypes, this multivariate genetic architecture suggests the usefulness of a phenome-wide approach to investigate the links between GPS and behavior, which is the focus of the next and final section of results.

### GPS correlate with the behavioral phenome

Figure 1 summarizes correlations between the 50 traits of the behavioral phenome and the 13 GPS for *P*_T_=0.30. Correlation coefficients, s.e., *P*-value thresholds (*P*_T_), and number of SNPs included are shown in Supplementary Table 3 for the fixed *P*_T_ (0.30; 0.10; 0.05). Very similar patterns of association emerged from both the conventional fixed *P*_T_ analyses and the high-resolution analyses that estimate the *P*_T_ flexibly for the ‘best-fit' GPS (Supplementary Figure 2 and Supplementary Table 4). Both methods yielded statistically significant phenomic associations only for the GPS for College and Child IQ.

#### College GPS

College GPS, which was based on the binary measure of attending college or not, showed the strongest phenomic profile at age 16, which might reflect the fact that its meta-analytic GWAS sample size was one of the largest (*N*=120 000; Rietveld *et al.*^18^). College GPS correlated significantly with academic performance at age 16: General Certificate of Secondary Examination (GCSE) English (*r* =0.15), GCSE mathematics (*r* =0.15, s.e. 0.02) and GCSE science (*r* =0.14, s.e. 0.02).^39^ College GPS also correlated significantly with general cognitive ability (‘g') (*r*=0.14, s.e. 0.03) as well as its subscales Ravens Matrices (*r*=0.12, s.e. 0.03) and with Mill Hill Vocabulary (*r*=0.09, s.e. 0.03), which confirms a similar finding for adults.^40^ College GPS also correlated positively with PISA math interest (*r*=0.10, s.e. 0.03) and math self-efficacy (*r*=0.12, s.e. 0.03). Negative associations for College GPS emerged for SDQ total behavior problems (*r*=−0.07, s.e. 0.02) and SDQ Conduct (*r*=−0.08, s.e. 0.02).

#### Child IQ GPS

The GPS for Child IQ yielded a similar but diluted phenomic profile as compared with College GPS. Child IQ GPS correlated significantly with GCSE English (*r*=0.09, s.e. 0.02), GCSE Math (*r*=0.10, s.e. 0.02) and GCSE Science (*r*=0.09, s.e. 0.02).

#### Psychiatric GPS

In contrast, the five psychiatric GPS yielded no significant correlations that passed multiple comparisons corrections across the behavioral phenome. Nominally significant associations included a positive correlation between Alzheimer's GPS and Conner's Impulsivity; and positive associations between Autism Spectrum Disorder GPS and Autism Quotient: Attention Switching. Autism Spectrum Disorder GPS yielded nominally significant negative associations with Chaos at home, Attachment and Height. Schizophrenia GPS correlated positively with GCSE English and negatively with Autism Quotient: Attention to Detail. Bipolar disorder GPS correlated negatively with Autism Quotient: Attention to Detail.

One likely explanation for the lower phenomic profile of psychiatric GPS compared with that of College GPS is the difference in sample sizes for the discovery samples. However, Child IQ GPS yielded significant associations despite the relatively smaller sample size of the GWAS (*N*=9616). This might point to the importance of developmental proximity or similarity of the phenotypes in discovery and target sample. It also emphasizes that predictive power is not only a function of sample size of the discovery sample.^5^ Phenotypic similarity between the traits in the discovery sample and the target sample is a proxy for the magnitude of genetic covariance between the traits.

The underlying premise of GWAS is that the polygenic architecture of complex traits and common disorders requires a genome-wide approach despite the multiple testing burden. Similarly, based on strong evidence for the ubiquitous pleiotropy of complex traits,^7, 9, 10, 11, 12, 13, 34^ the advantage of the phenome-wide approach outweighs the resulting multiple testing burden. Specifically, while testing a large number of highly unlikely hypotheses with little or no prior support should be avoided, in this case we have collated a well-defined set of psychological and behavioral traits for which there is good reason to suspect causal associations with the available discovery GWAS phenotypes. In this way, the only 'multiple testing problem' relates to setting an appropriate significance threshold given the number and correlation of tests performed (see Supplementary Methods for multiple testing correction method used).

Therefore, the absence of phenome-wide significant associations (that is, after correcting for multiple testing across the 50 traits and 13 GPS) for all psychiatric GPS does not imply the absence of polygenic effects. However, the scarcity of nominally significant associations between the psychiatric GPS and the 50 traits suggests that the genetic covariance between psychiatric adult case/control samples and our adolescent population sample might be relatively small. For instance, under certain assumptions about polygenic architecture (for example, ⩽5% of tested SNPs associated with schizophrenia in the discovery GWAS), we had ⩾80% power with α=0.05 to detect associations between the Schizophrenia GPS and a phenotype given ⩾0.06 genetic covariance between schizophrenia and the target trait, with ⩾0.5% of phenotypic variation in the target trait explained by schizophrenia^5, 41, 42^ (see Supplementary Methods for more detail).

One possible reason for the lower observed phenomic profile of the psychiatric GPS might be that the current sample is UK representative and therefore not enriched for psychiatric symptoms. The psychiatric GPS were based on case–control comparisons, often with extreme cases. This emphasizes the limitations of using GPS for the prediction of trait variation in the general population from GWAS based on selected samples. Importantly, the GPS College did predict children's behavior problems in our UK-representative sample, whereas the psychiatric GPS did not. This points to the usefulness of cross-trait prediction in general and the value of cognitive GWAS/GPS as prediction instruments for psychiatric symptoms in the population.

#### Other GPS

Adult body mass index (BMI) GPS correlate positively with the measure of BMI at age 16 (*r*=0.18, s.e. 0.03); and adult Height GPS correlate with height at age 16 (*r*=0.33, s.e. 0.03). There was suggestive evidence for a negative association between Ever smoked GPS and conscientiousness (*r*=−0.06, s.e. 0.03) and a positive association with BMI (*r*=0.09, s.e. 0.03).

### Quantile analyses

**Figure 2:**
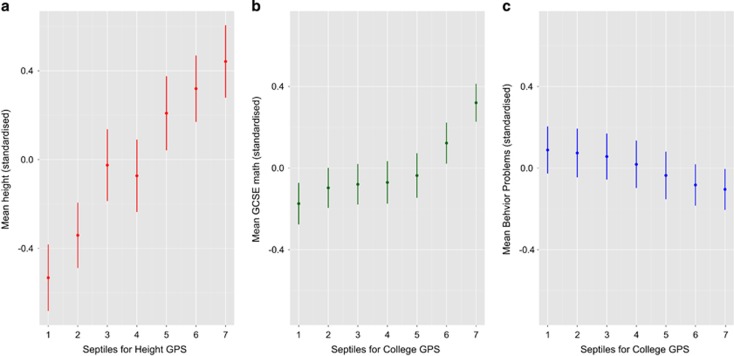
(**a**) Mean for height at age 16 by adult Height genome-wide polygenic score (GPS) septile. The threshold for selecting trait-associated alleles was *P*_T_ < 0.30. The GPS were converted to quantiles (1=lowest, 7=highest GPS). Mean phenotypic values and 95% confidence intervals (CIs) for the quantile groups (bars) were estimated using general linear regression with ancestrally informative principal components, sex and age of measurement as covariates. (**b**) Mean for children's mathematics educational achievement at age 16 (compulsory subject on the General Certificate of Secondary Examination (GCSE), see Materials and Methods for details) by College GPS septile. The threshold for selecting trait-associated alleles was *P*_T_ < 0.30. The GPS were converted to quantiles (1=lowest, 7=highest GPS). Mean phenotypic values and 95% CI for the quantile groups (bars) were estimated using general linear regression with ancestrally informative principal components, sex and age of measurement as covariates. (**c**) Mean for total parent-reported behavior problems at age 16 by adult College GPS septile. The threshold for selecting trait-associated alleles was *P*_T_ < 0.30 (the best-fit GPS as estimated by PRSice software, see Materials and Methods). The GPS were converted to quantiles (1=lowest, 7=highest GPS). Mean phenotypic values and 95% CI for the quantile groups (bars) were estimated using general linear regression with ancestrally informative principal components, sex and age of measurement as covariates.

To illustrate the ability to stratify individuals by GPS and the potential of polygenic score for phenotype prediction, we grouped individuals into GPS septiles and estimated the mean phenotypic value for each quantile. We provide three examples:

Figure 2a shows that mean standardized height increased with more adult height-associated alleles in our UK-representative sample of children aged 16, with the largest difference between the lowest and highest septile (Hedges *g:* −1.01 with 95% confidence interval (CI): −1.26 to −0.77; difference in means: 0.97 s.d., with *P*-value <0.01). Figure 2b shows that mean math grade on the standardized UK-national examinations at age 16 increased with more College-associated alleles (Figure 2b), with the largest difference between the lowest and highest septile (Hedges *g*: −0.52 with 95% CI: −0.67 to −0.37; difference in means: 0.49 s.d., with *P*-value <0.01). Figure 2c illustrates the utility of the phenome-wide approach for cross-trait prediction: the mean for total parent-reported behavior problems at age 16 decreased slightly but significantly with higher College GPS, with a maximum effect size between the lowest and highest quantile (Hedges *g*: 0.20 with 95% CI: 0.04–0.34; difference in means: 0.19, with *P*-value 0.01).

These results (Figure 2) illustrate the ability to stratify individuals by GPS, which suggests opportunities for research, for example, selecting high and low GPS extreme individuals for intensive research such as neuroimaging that is unable to test large representative samples. However, we emphasize that the current predictive power and accuracy of GPS do not allow for their use as predictive tests.

## DISCUSSION

These results highlight the usefulness of a phenome-wide approach to examine behavioral profiles of associations with GPS even though current GPS account for only a few percent of variance or liability of their target trait. An interesting finding is that phenome-wide associations for cognitive GPS are stronger than for psychiatric GPS in our UK-representative sample of adolescents. For example, we found that GPS College, but none of the psychiatric GPS, predicted adolescent behavior problems, which demonstrates the usefulness of cross-trait predictions and the multivariate phenome-wide approach in general. However, this finding could be explained by differences in sample sizes, sampling methods (population versus case/control), and genetic architecture (for example, extent of covariance between discovery and target trait).

Finding significant associations for the Child IQ GPS, which is based on a small discovery sample, is a reminder that predictive power of GPS is not merely a function of sample size but also of the developmental proximity of the GWAS sample and the target GPS sample. As explained in the Introduction, we were interested in the extent to which GWAS in adult samples yield GPS that can predict genetic propensities—strengths as well as weaknesses—earlier in development, in this case in adolescence. However, GPS College is a trait assessed closer in age to the adolescents in our sample. In contrast, the psychiatric GPS were derived from GWAS studies of adults.

A larger issue is that extant GPS account for only a few percent of the phenomic variance in the target trait. However, we illustrate the research potential of polygenic stratification by quantile. Power and accuracy of GPS will improve as GWAS sample sizes increase. GPS that narrow the ‘hidden heritability' gap is what is needed most for phenome-wide analyses—and for all research harvesting the fruits of GWAS.
