# Supplementary figures and images for: Phenome-wide analysis of genome-wide polygenic scores
Source: Mol Psychiatry. 2015 Aug 25;21(9):1188–93. doi: 10.1038/mp.2015.126 (PMC4767701; doi:10.1038/mp.2015.126)

Correlations Genome-wide Polygenic Scores and phenotypes pT = 0.1

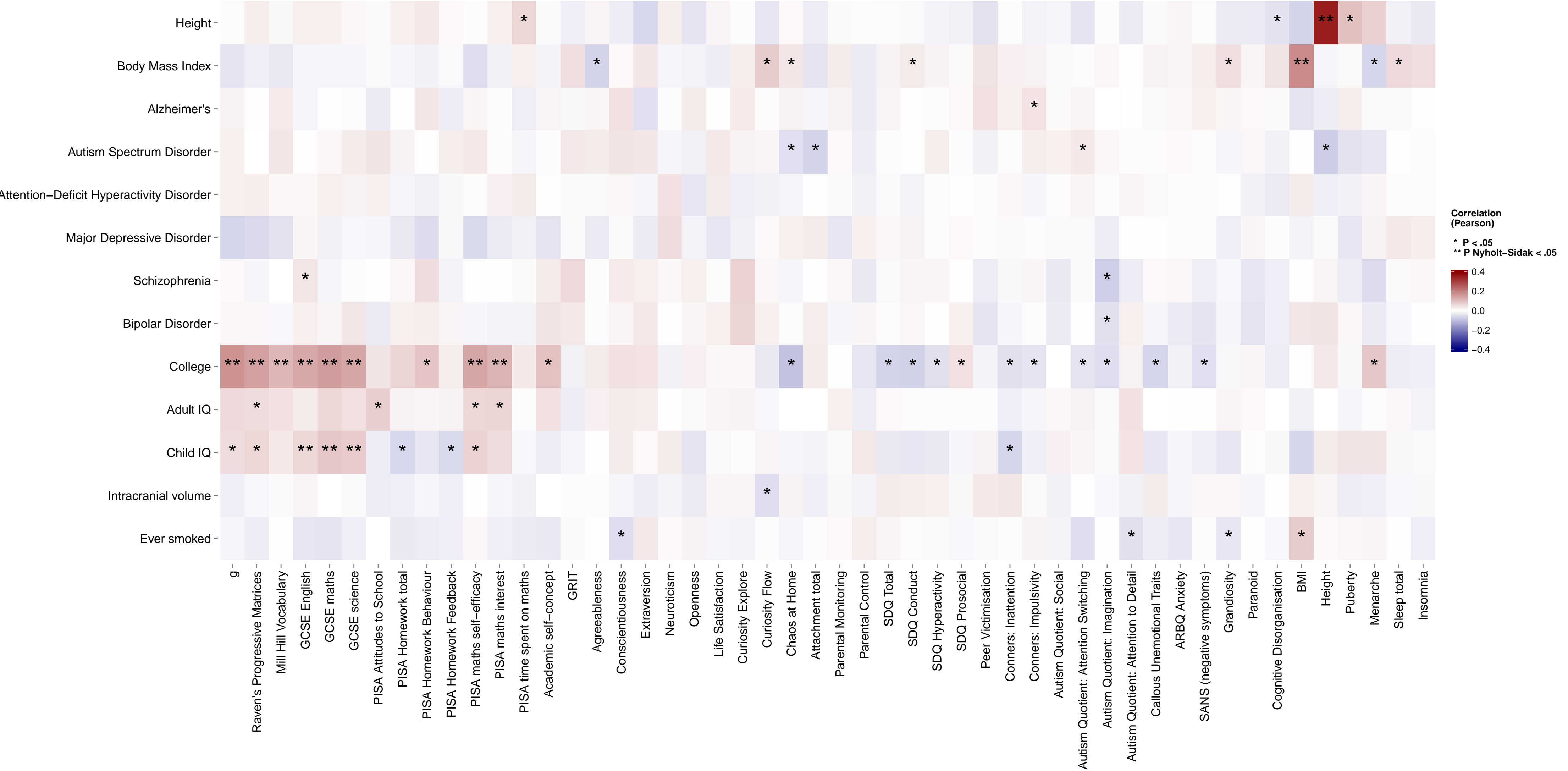

Supplement: Supplementary Figure 1a [file mp2015126x1.pdf]

Correlations Genome-wide Polygenic Scores and phenotypes pT = 0.05

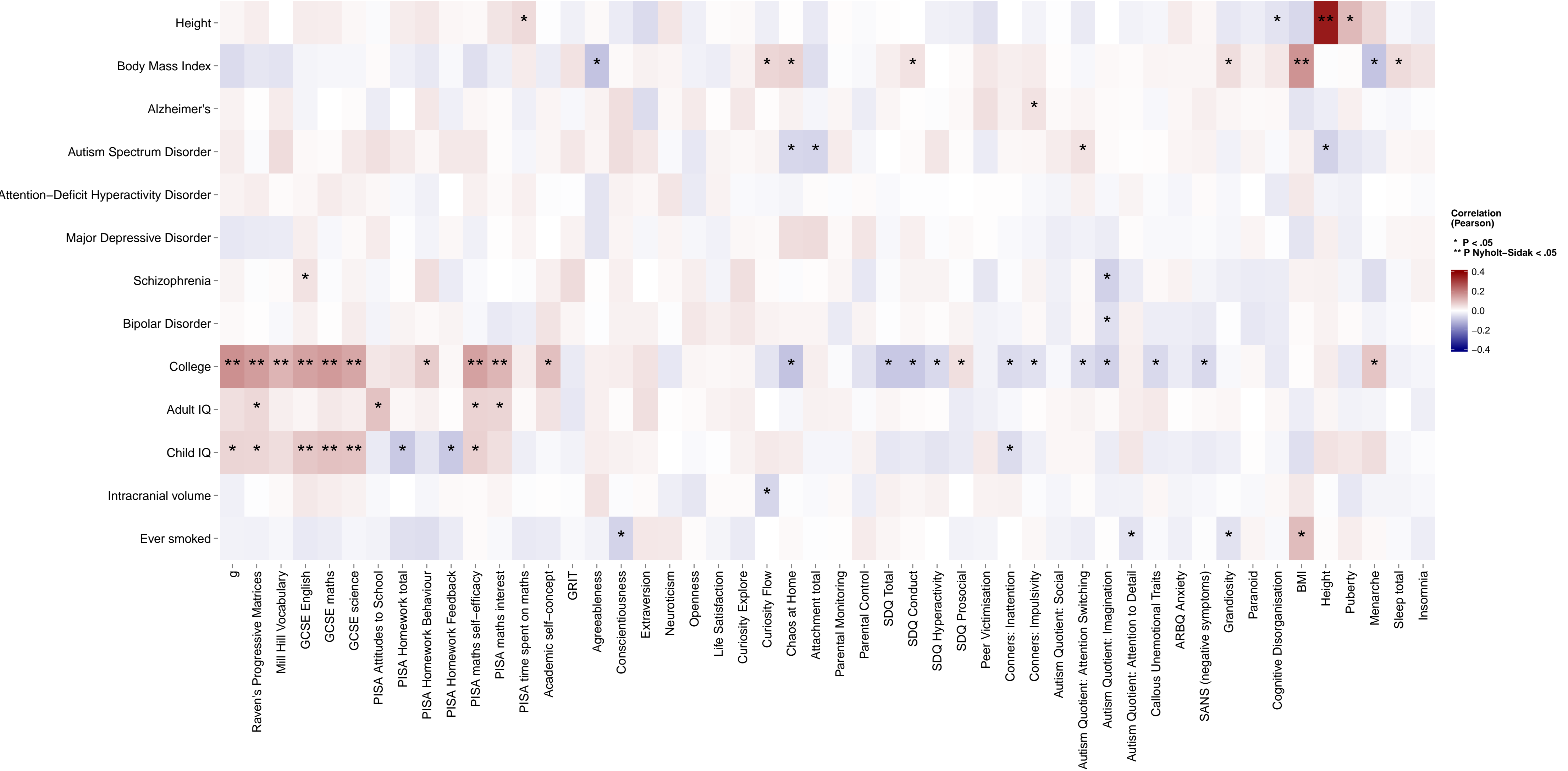

Supplement: Supplementary Figure 1b [file mp2015126x2.pdf]

### Correlations 'best-fit' Genome-wide Polygenic Scores and phenotypes

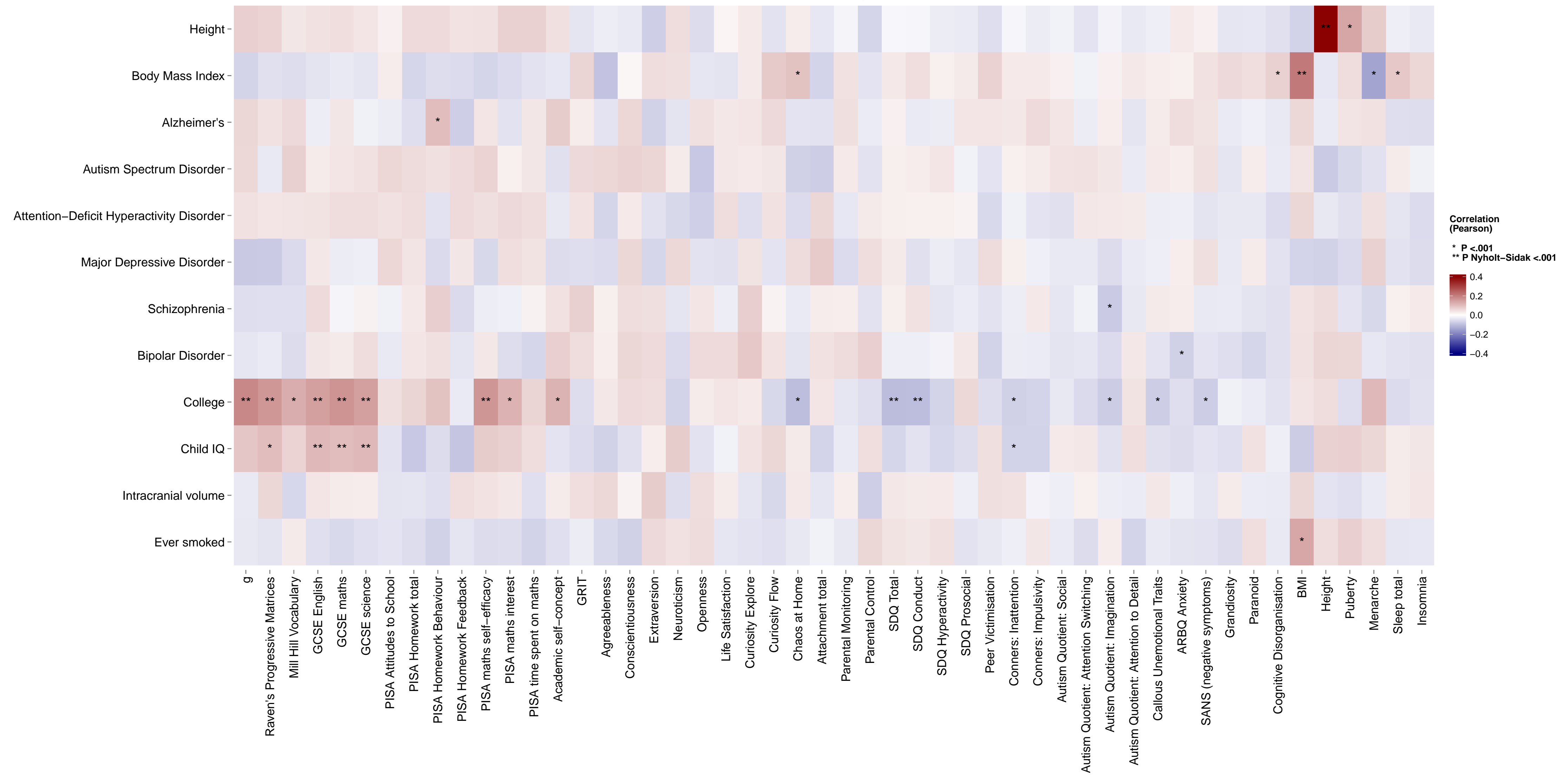

Supplement: Supplementary Figure 2 [file mp2015126x3.pdf]

Distributions Genome-wide Polygenic Scores at P-Value Threshold 0.30

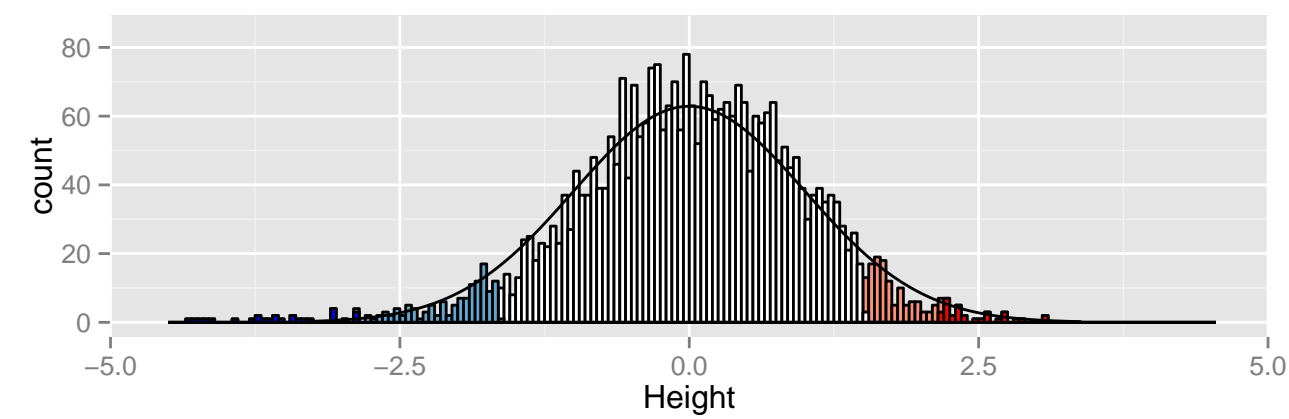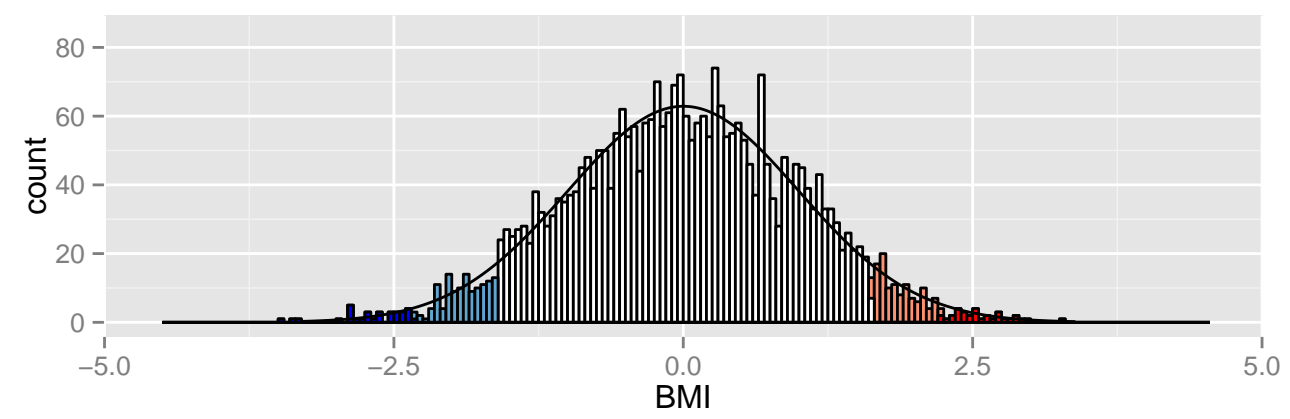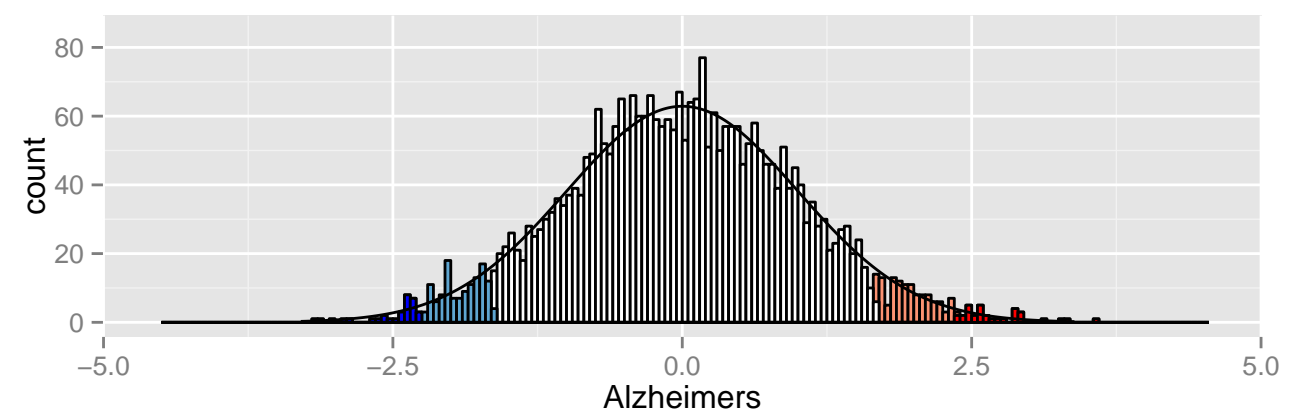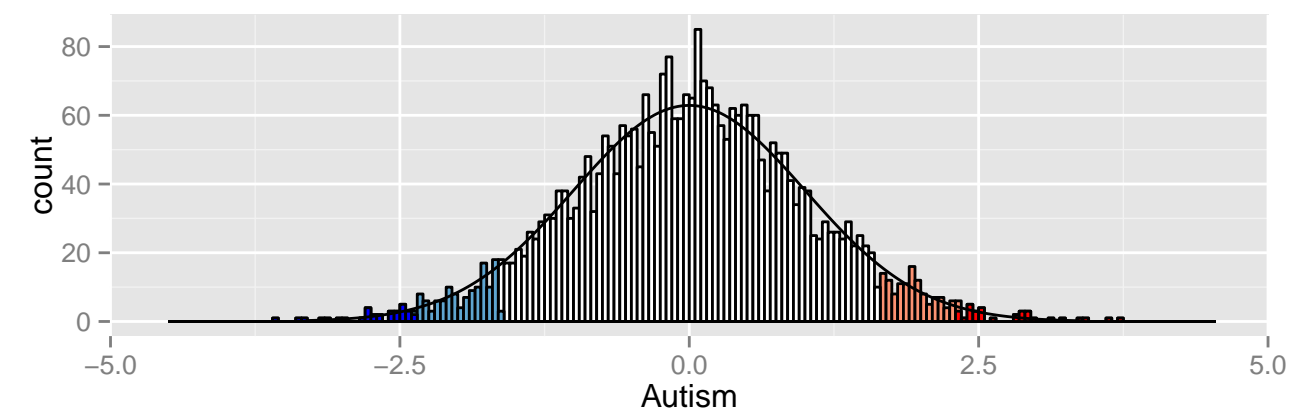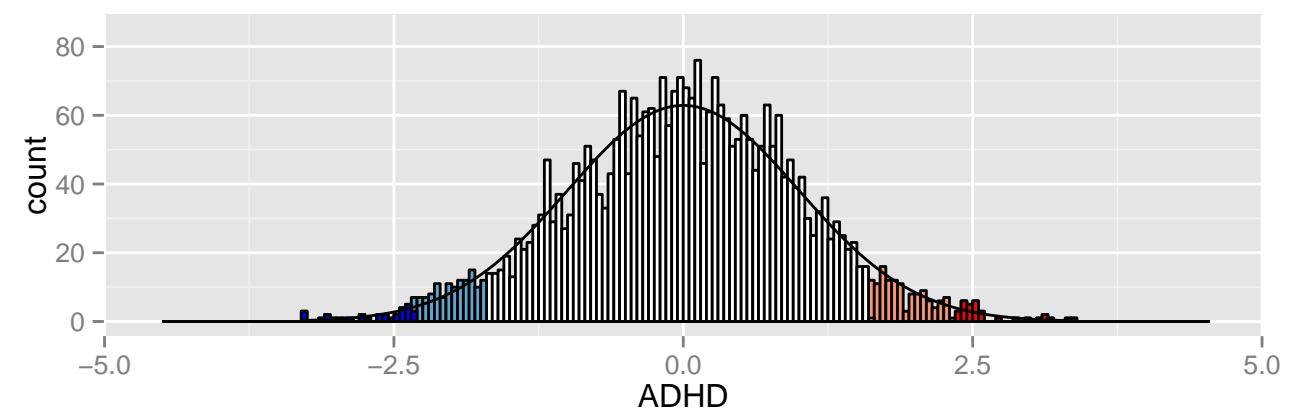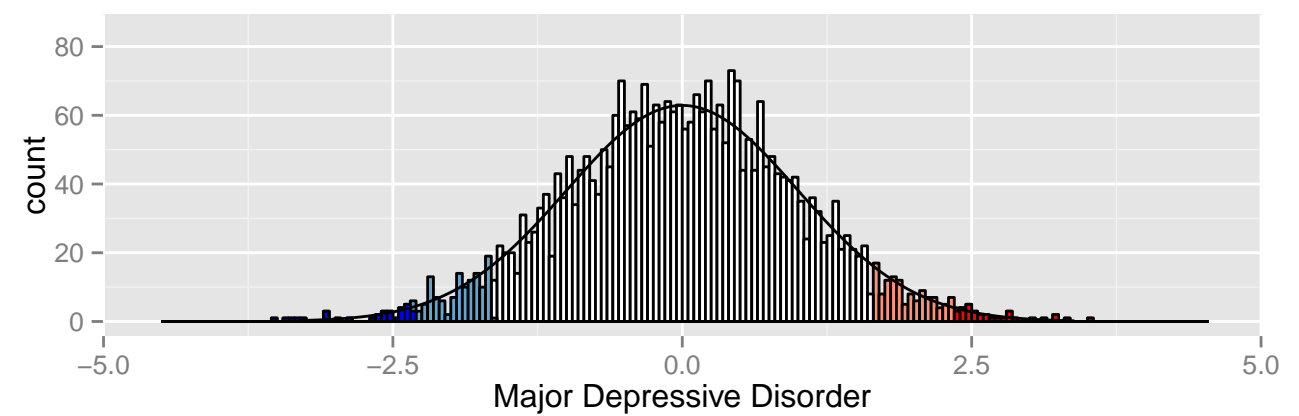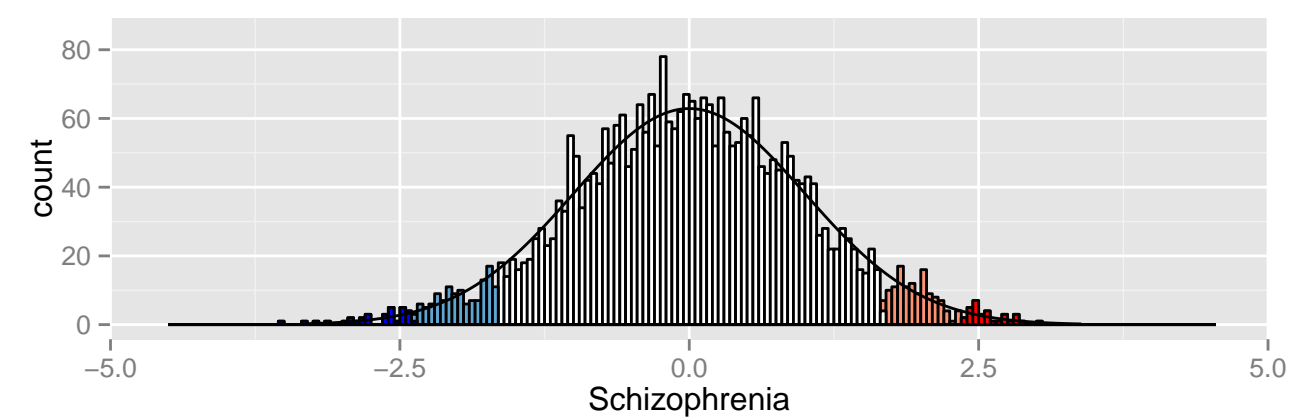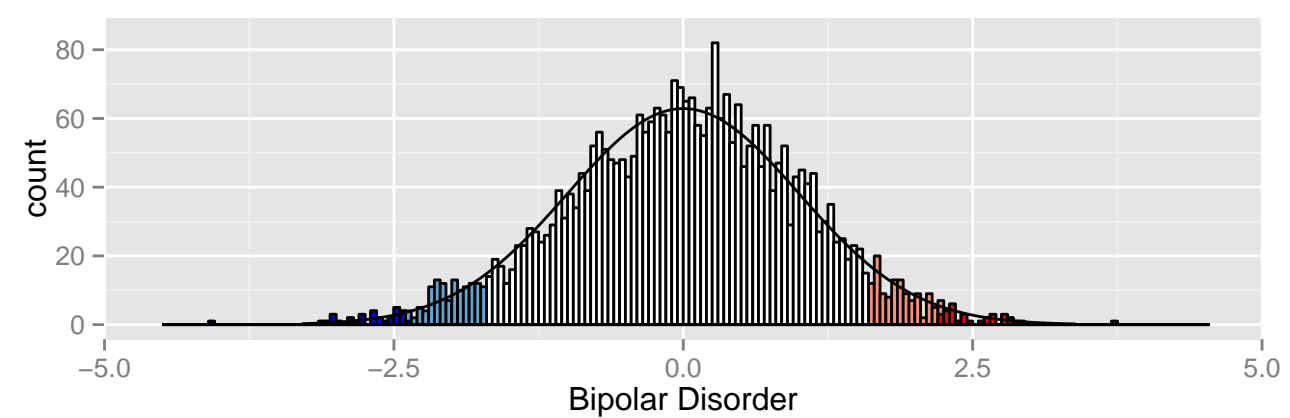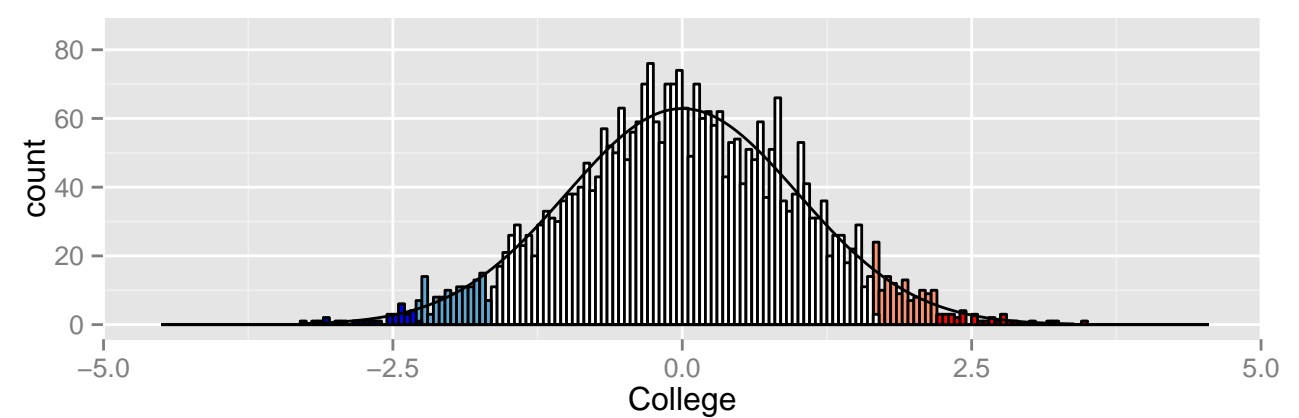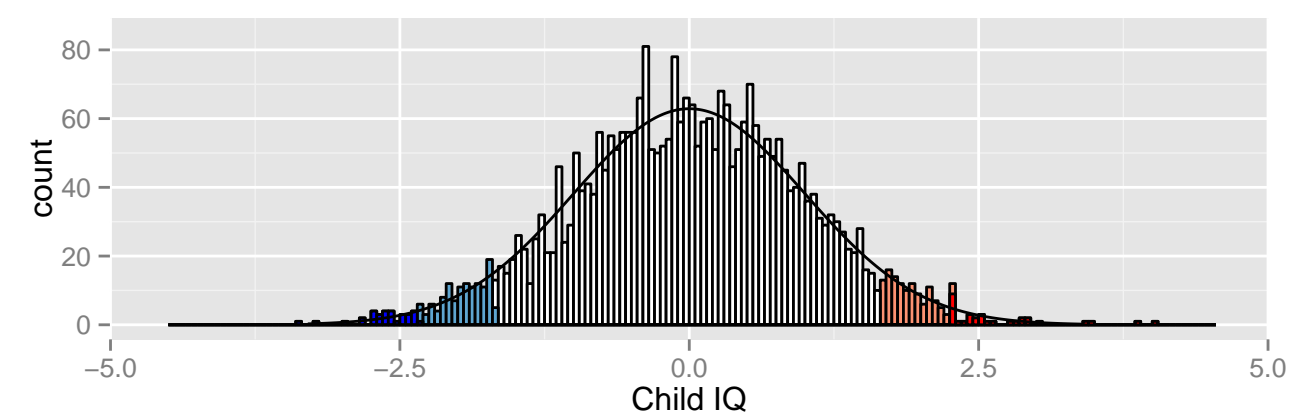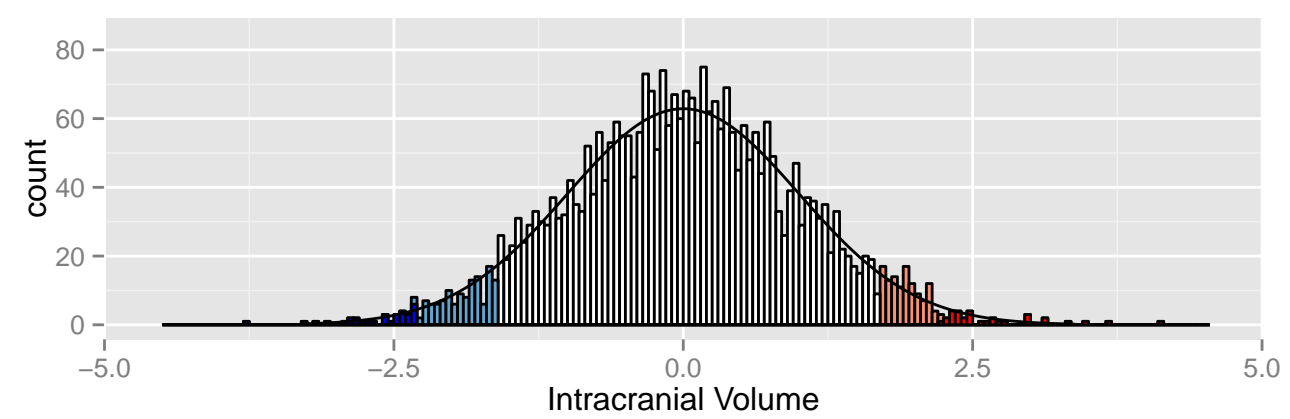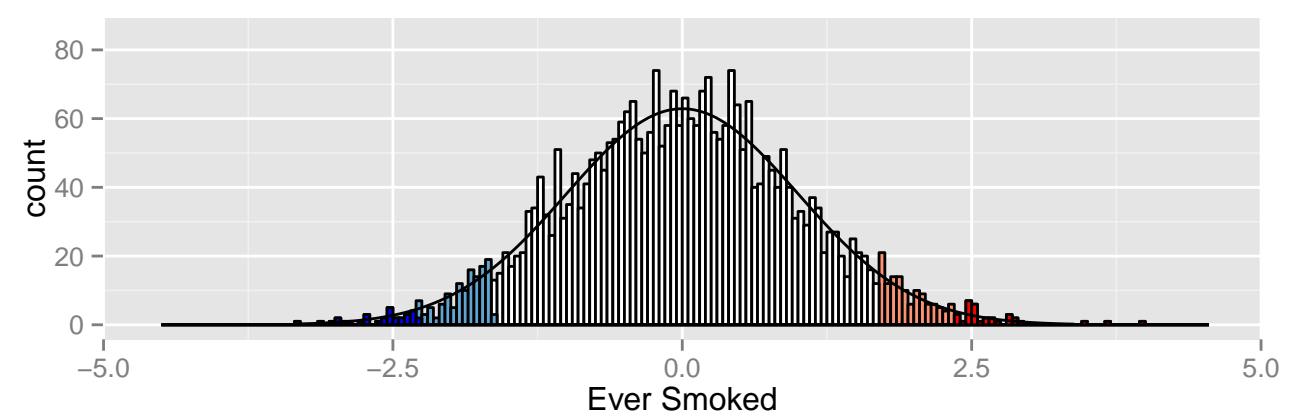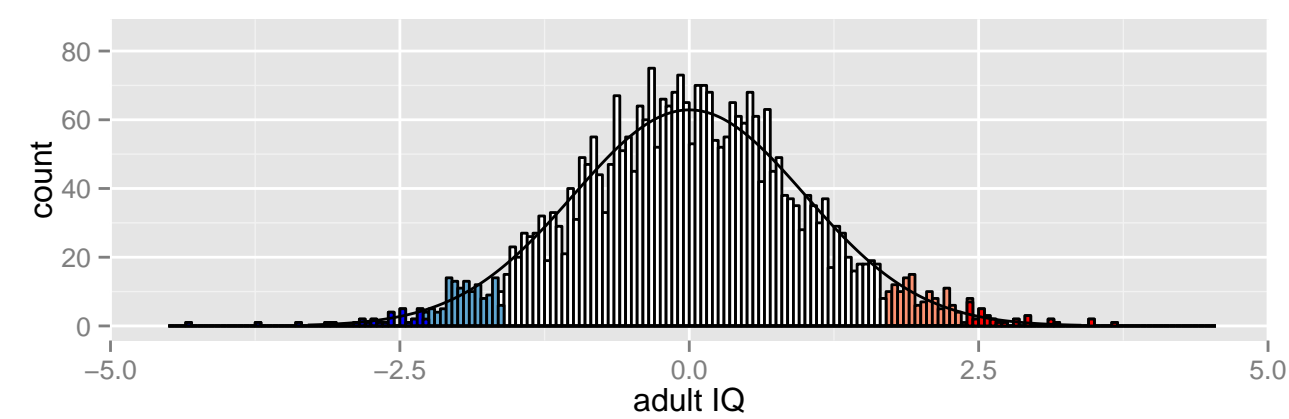

Supplement: Supplementary Figure 3a [file mp2015126x4.pdf]

Distributions Genome-wide Polygenic Scores at P-Value Threshold 0.10

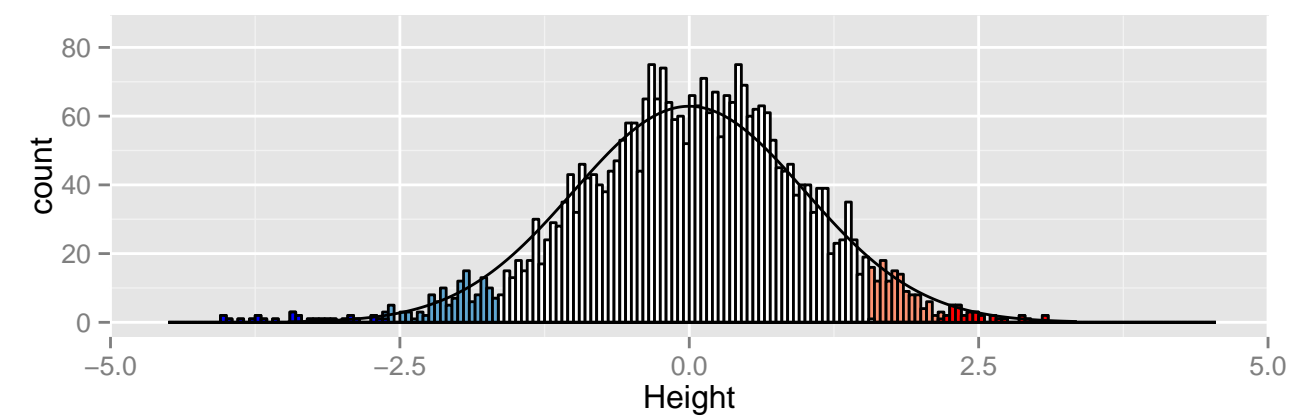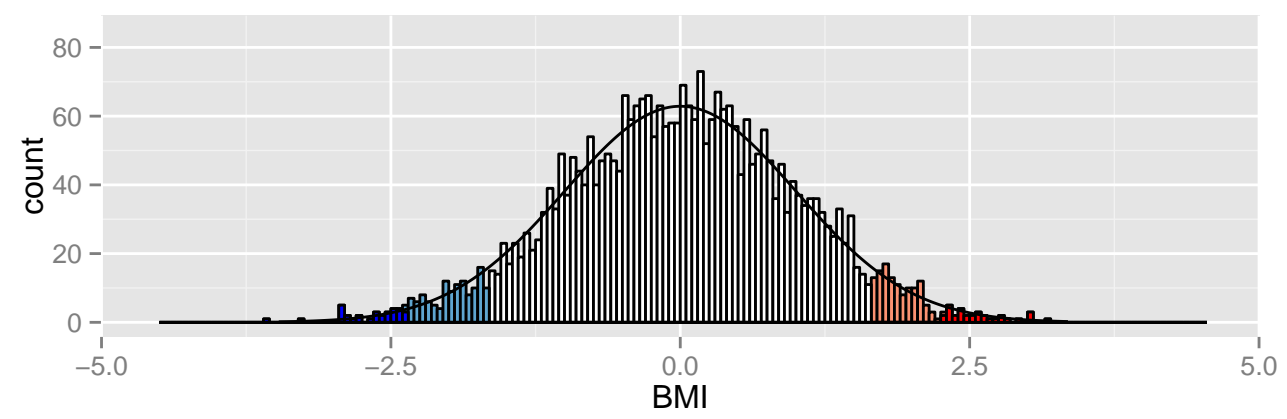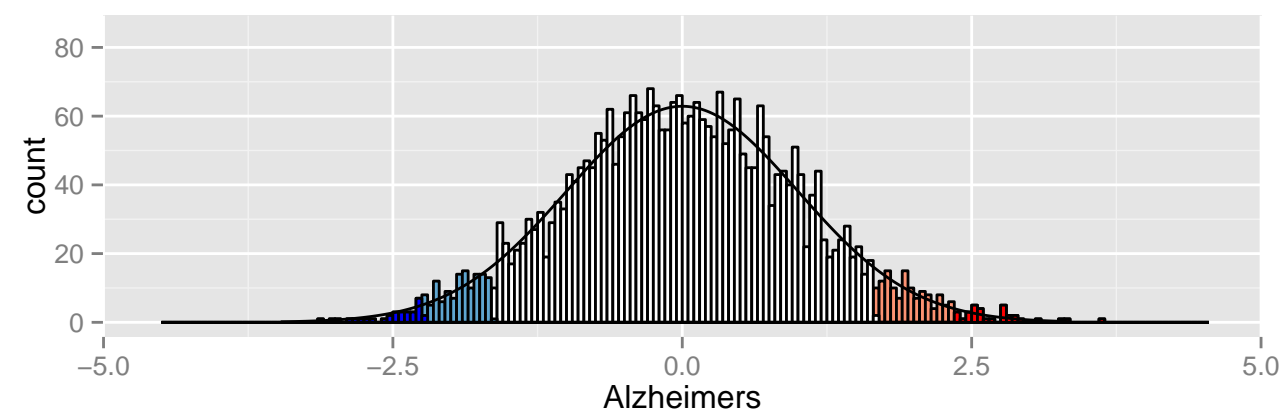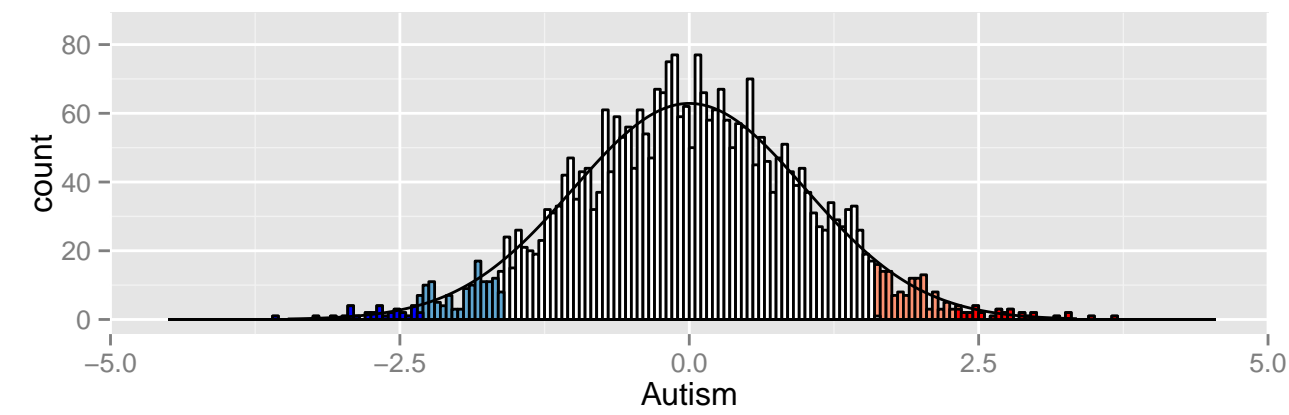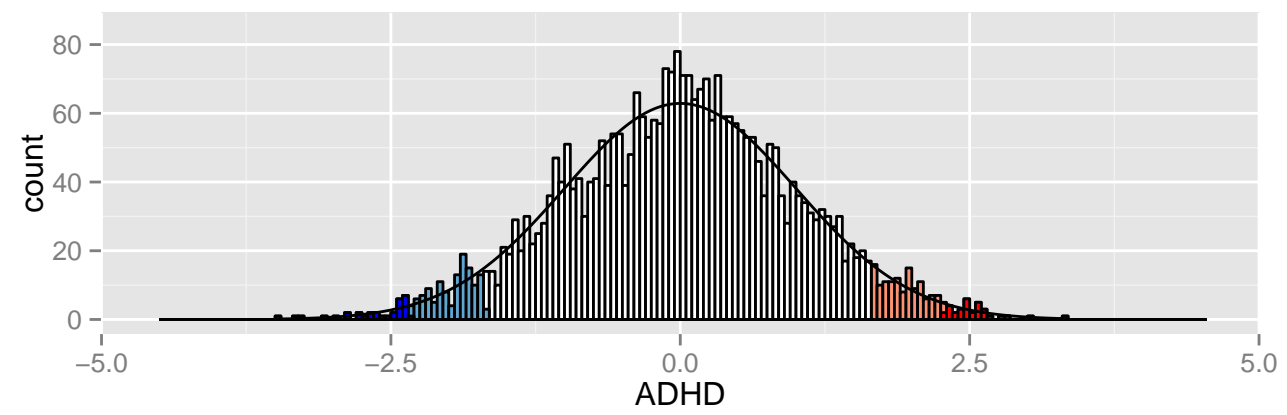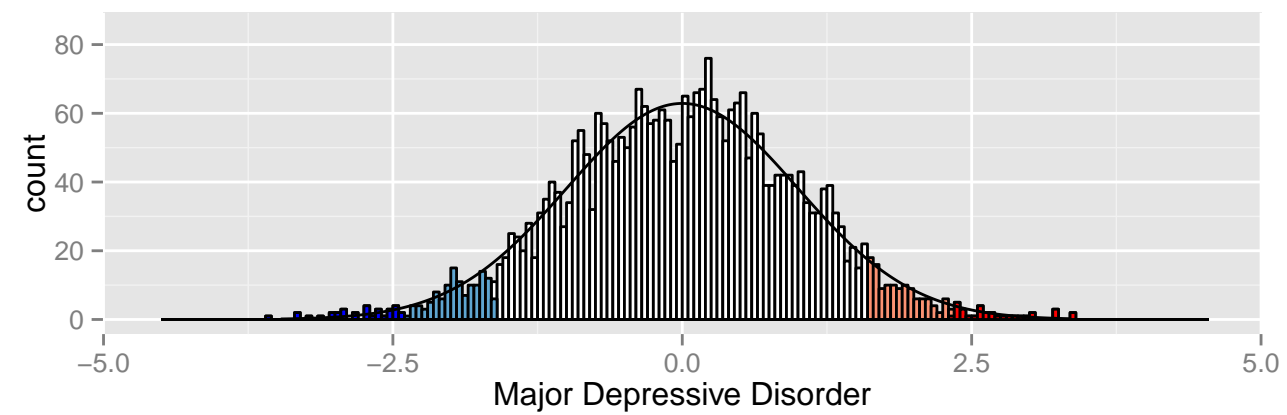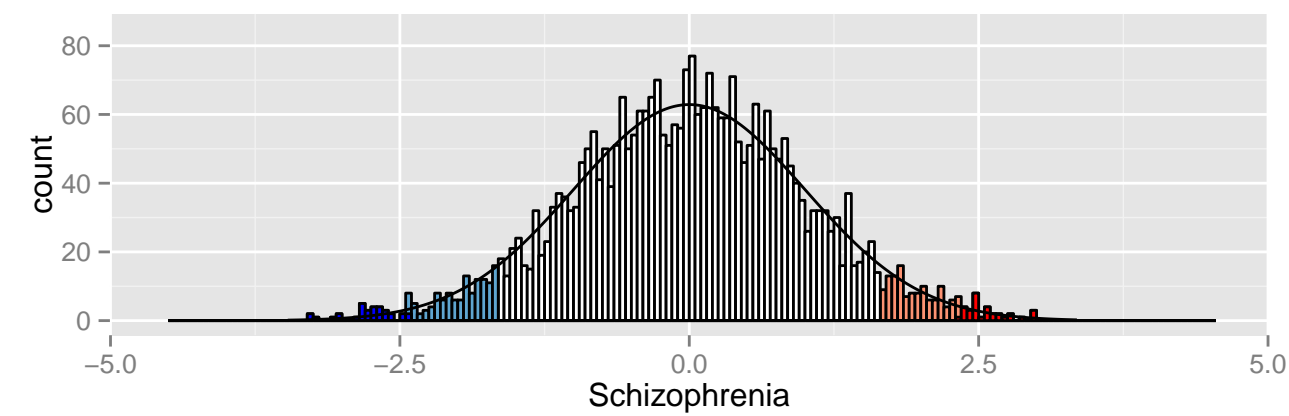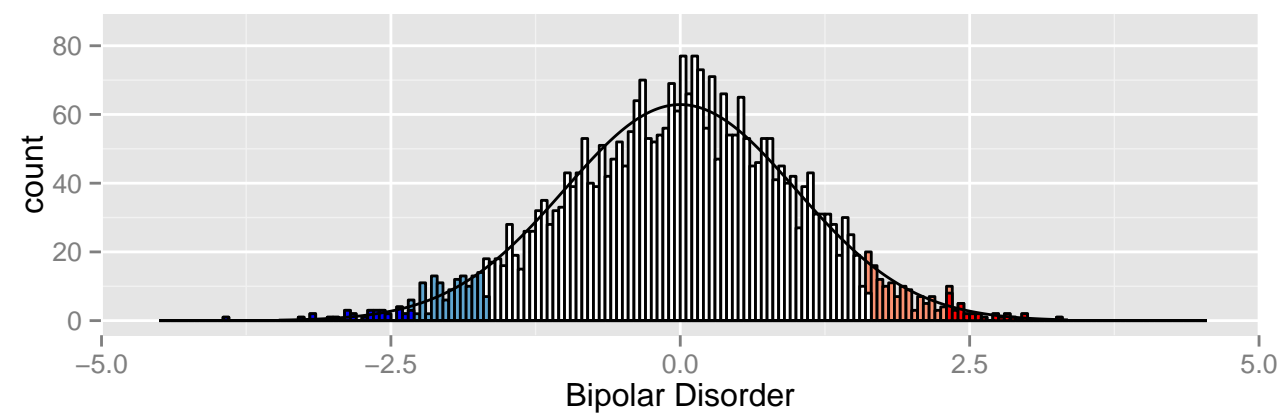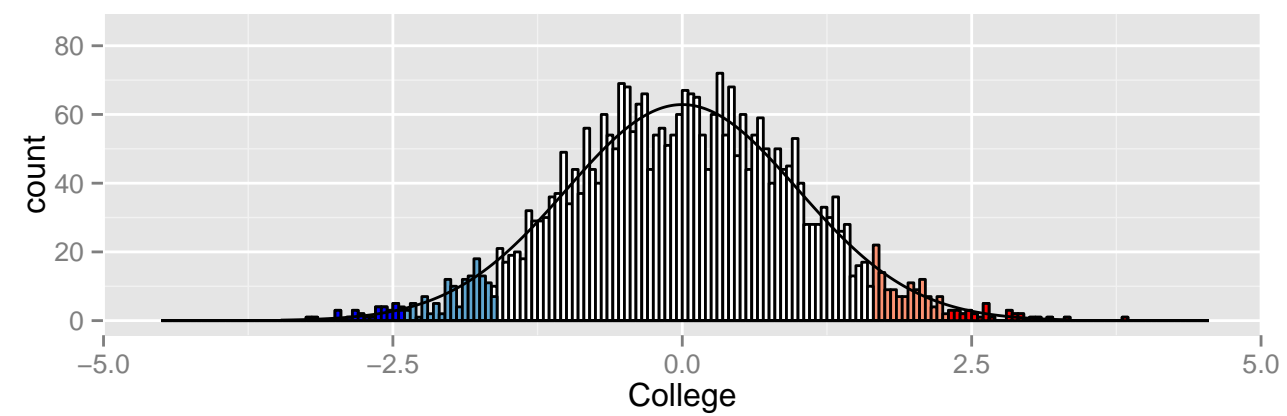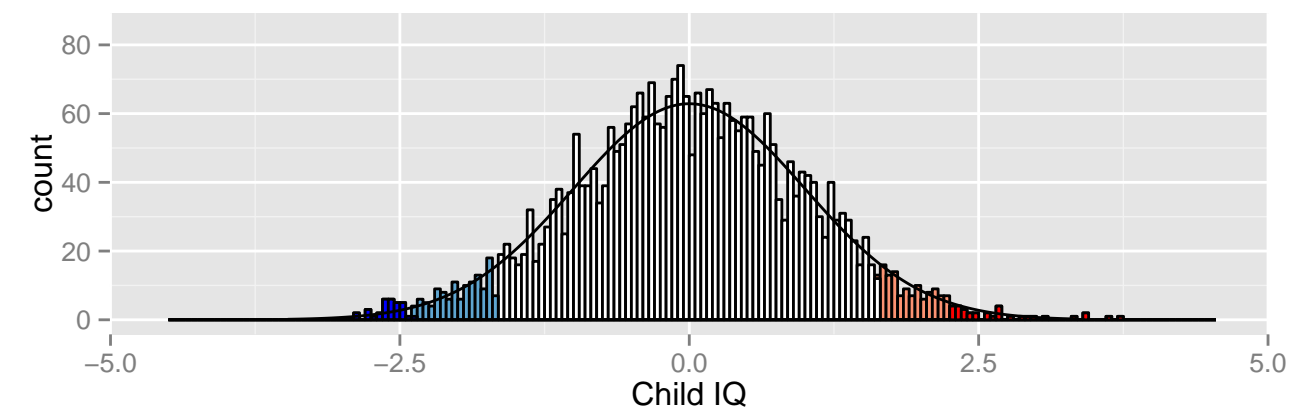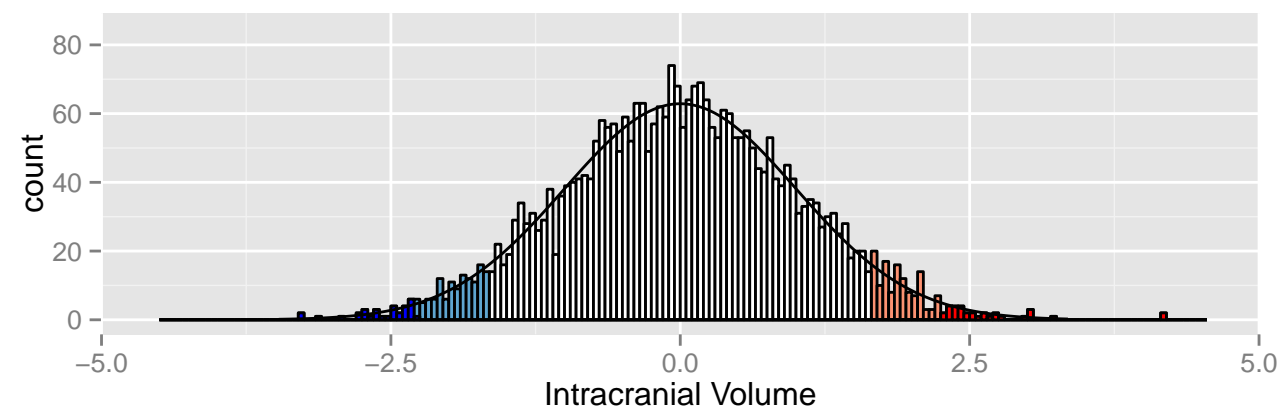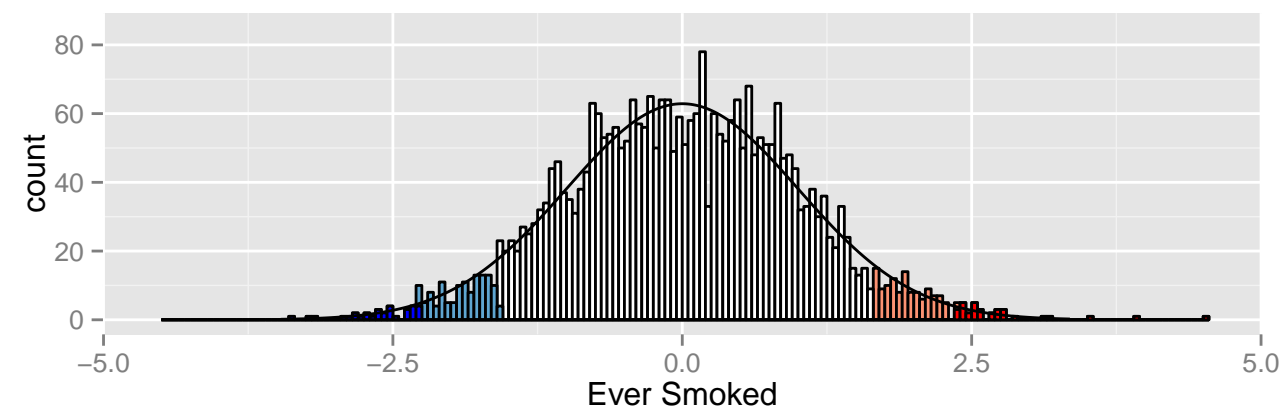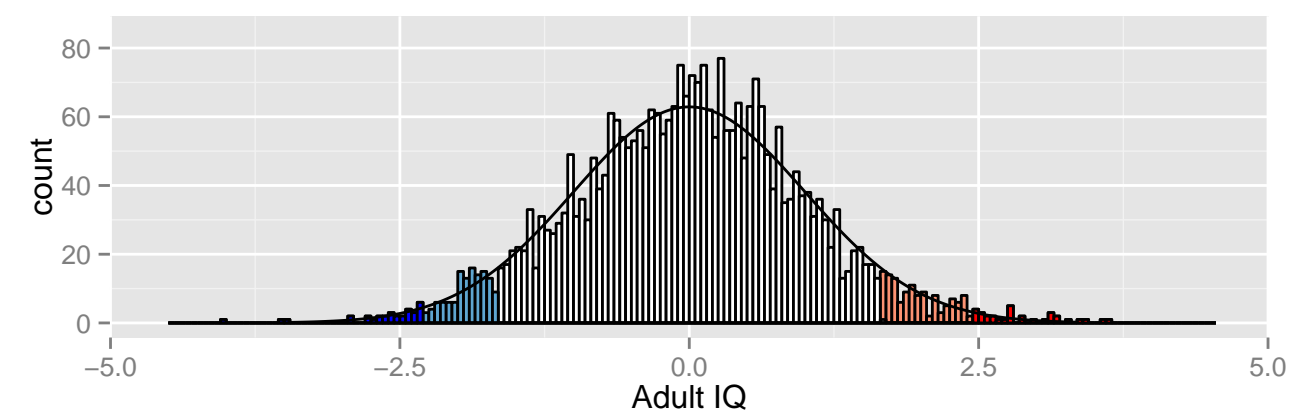

Supplement: Supplementary Figure 3b [file mp2015126x5.pdf]

Distributions Genome-wide Polygenic Scores at P-Value Threshold 0.05

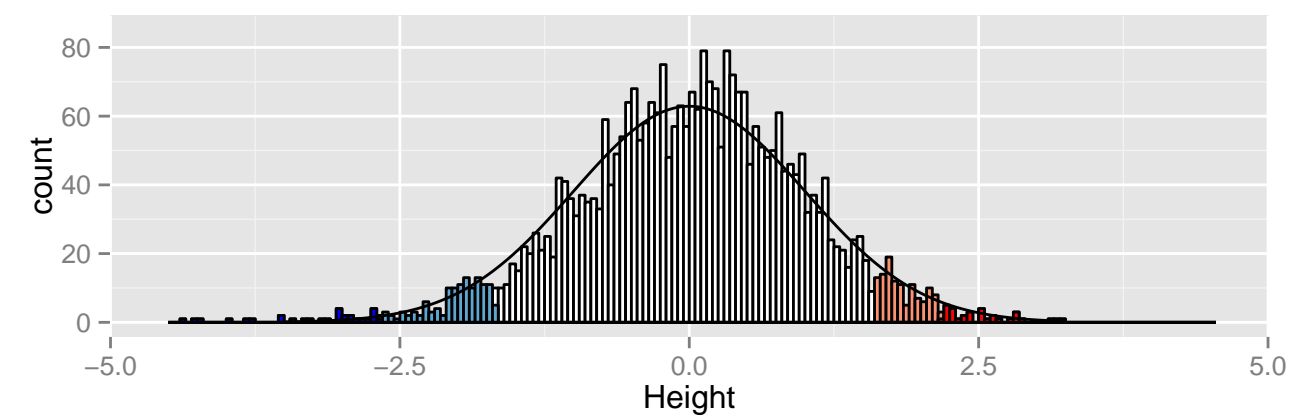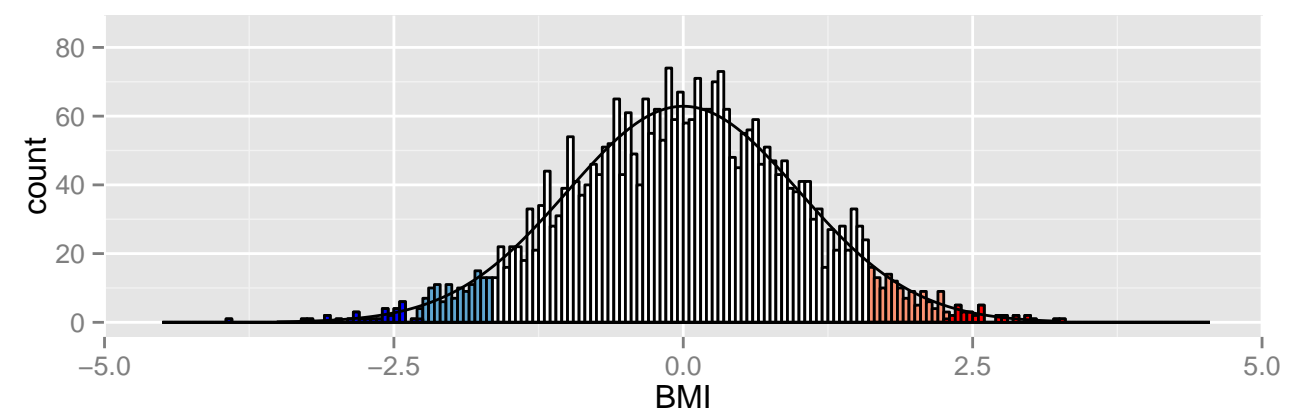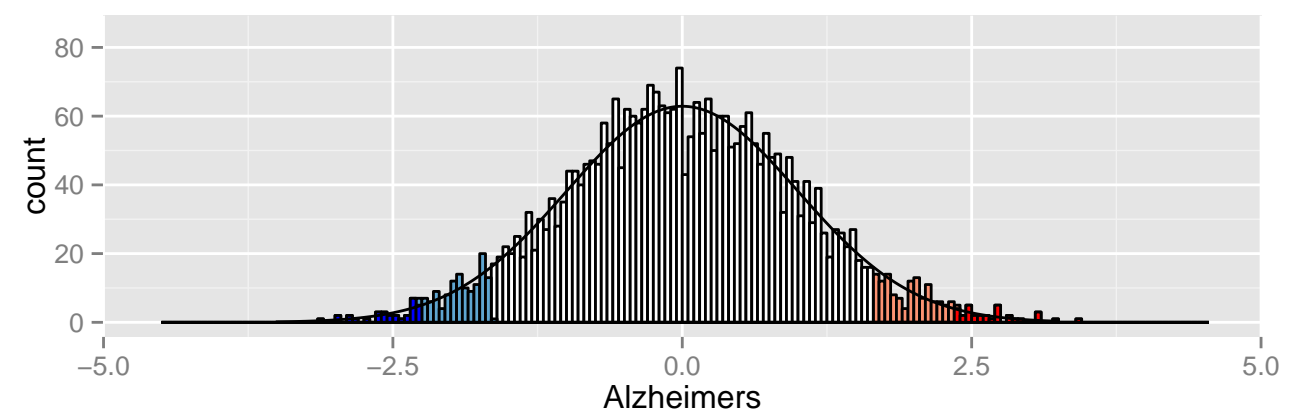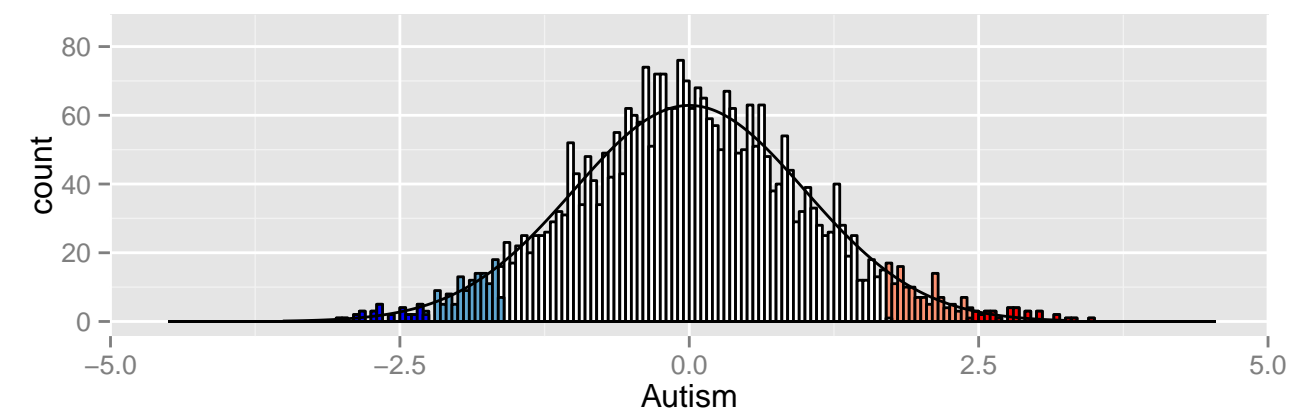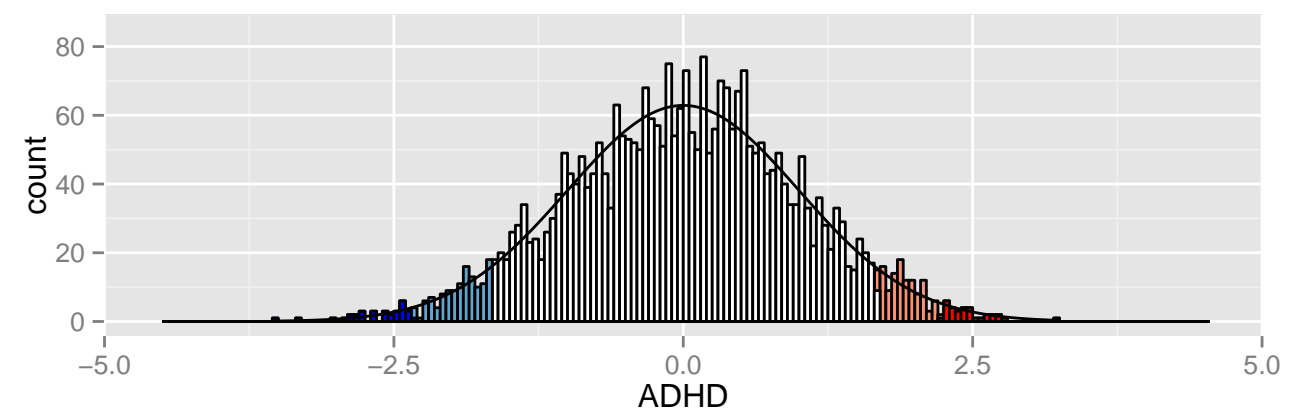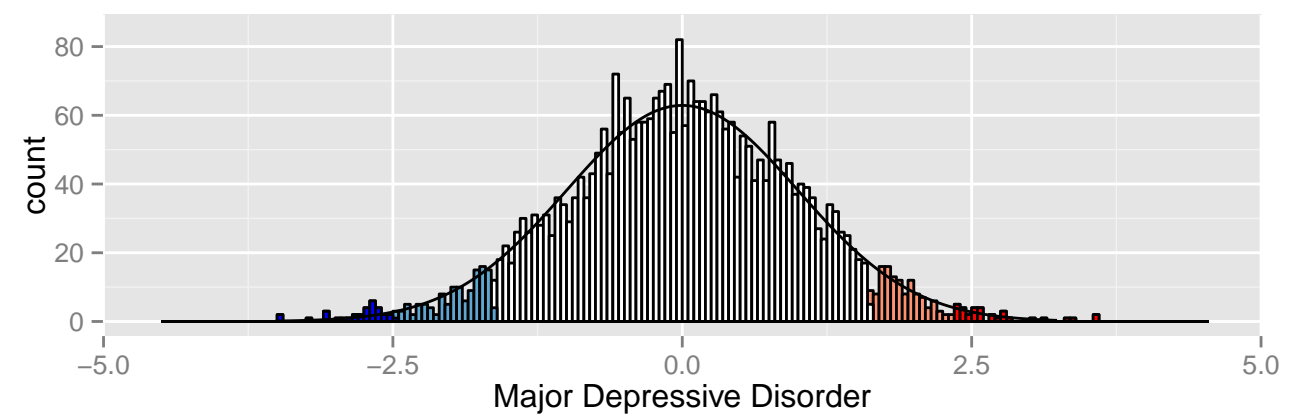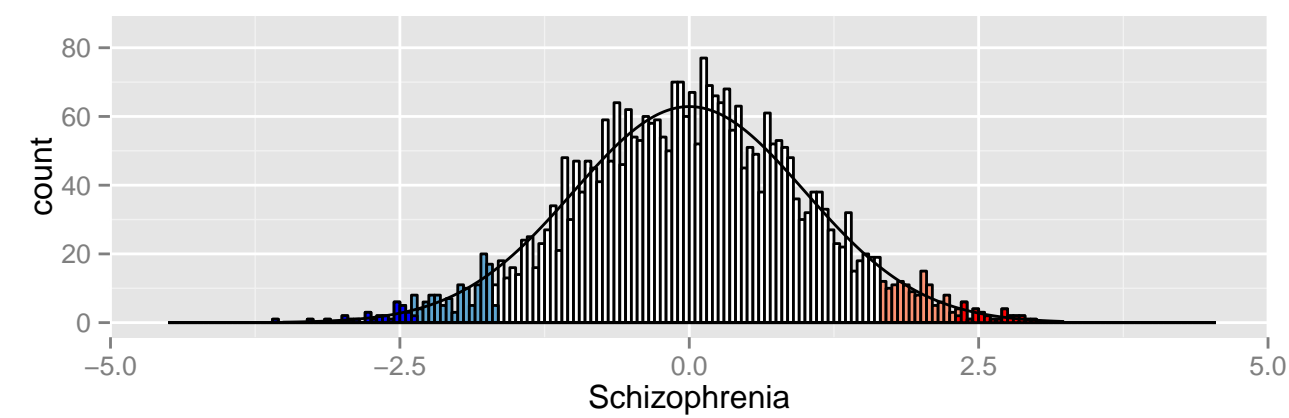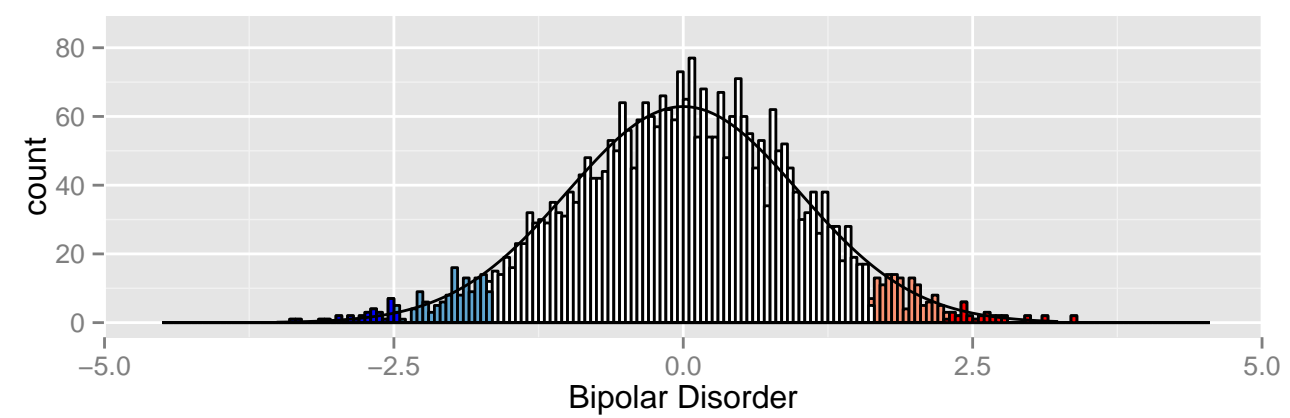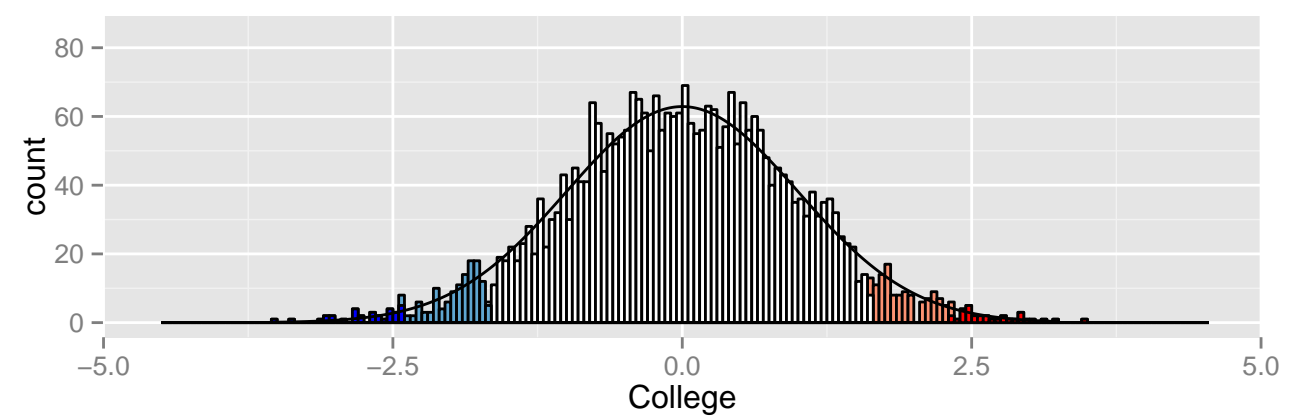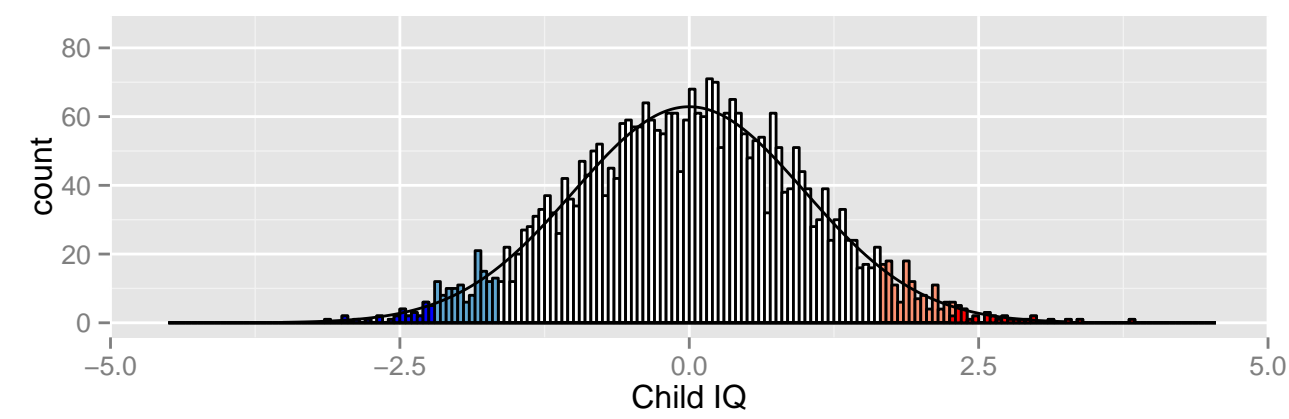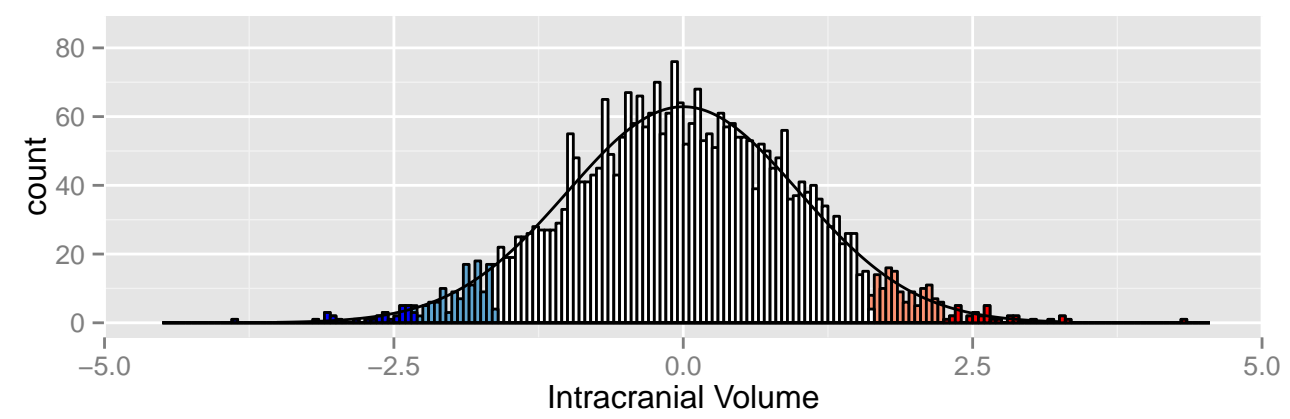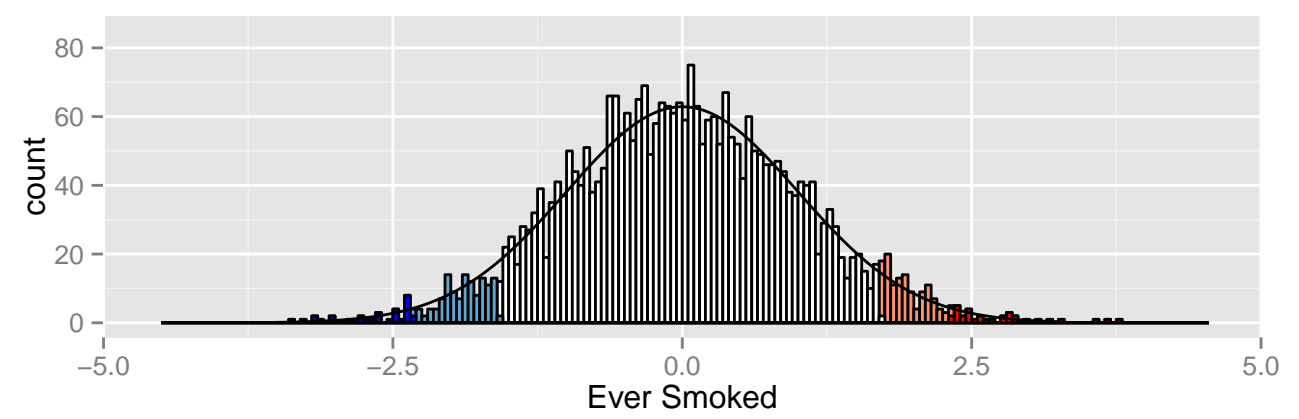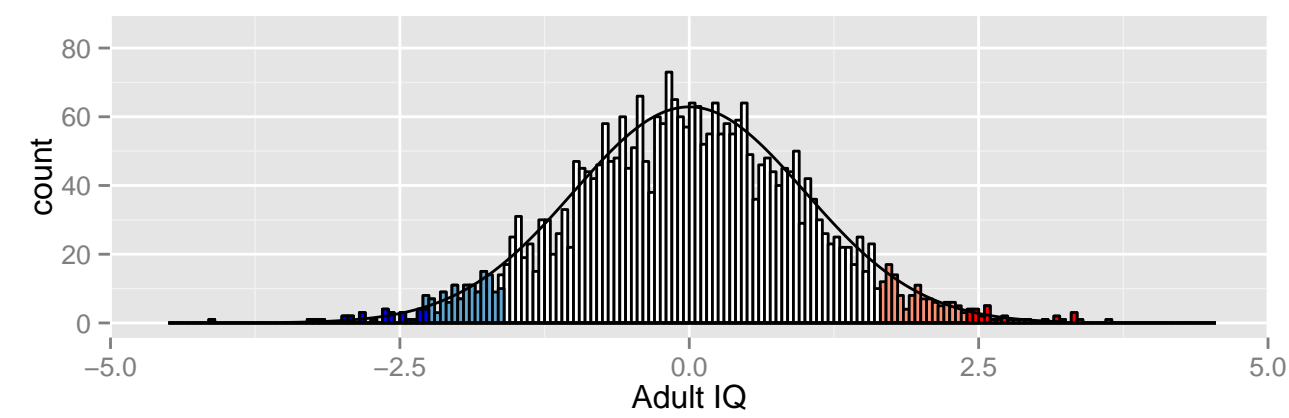

Supplement: Supplementary Figure 3c [file mp2015126x6.pdf]

Correlations between target phenotypes

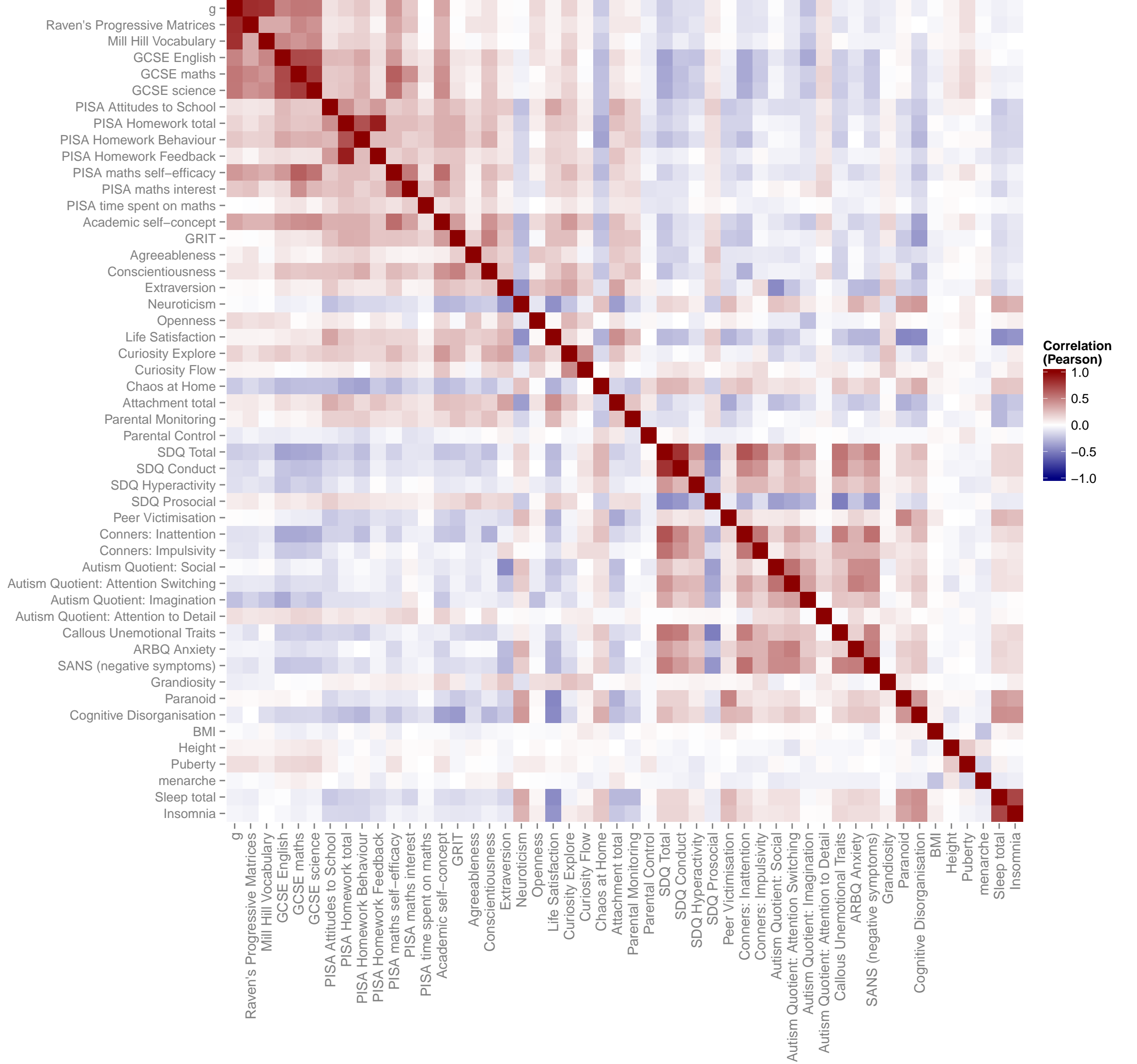

Supplement: Supplementary Figure 4 [file mp2015126x7.pdf]

Genetic Correlations among 13 GWAS

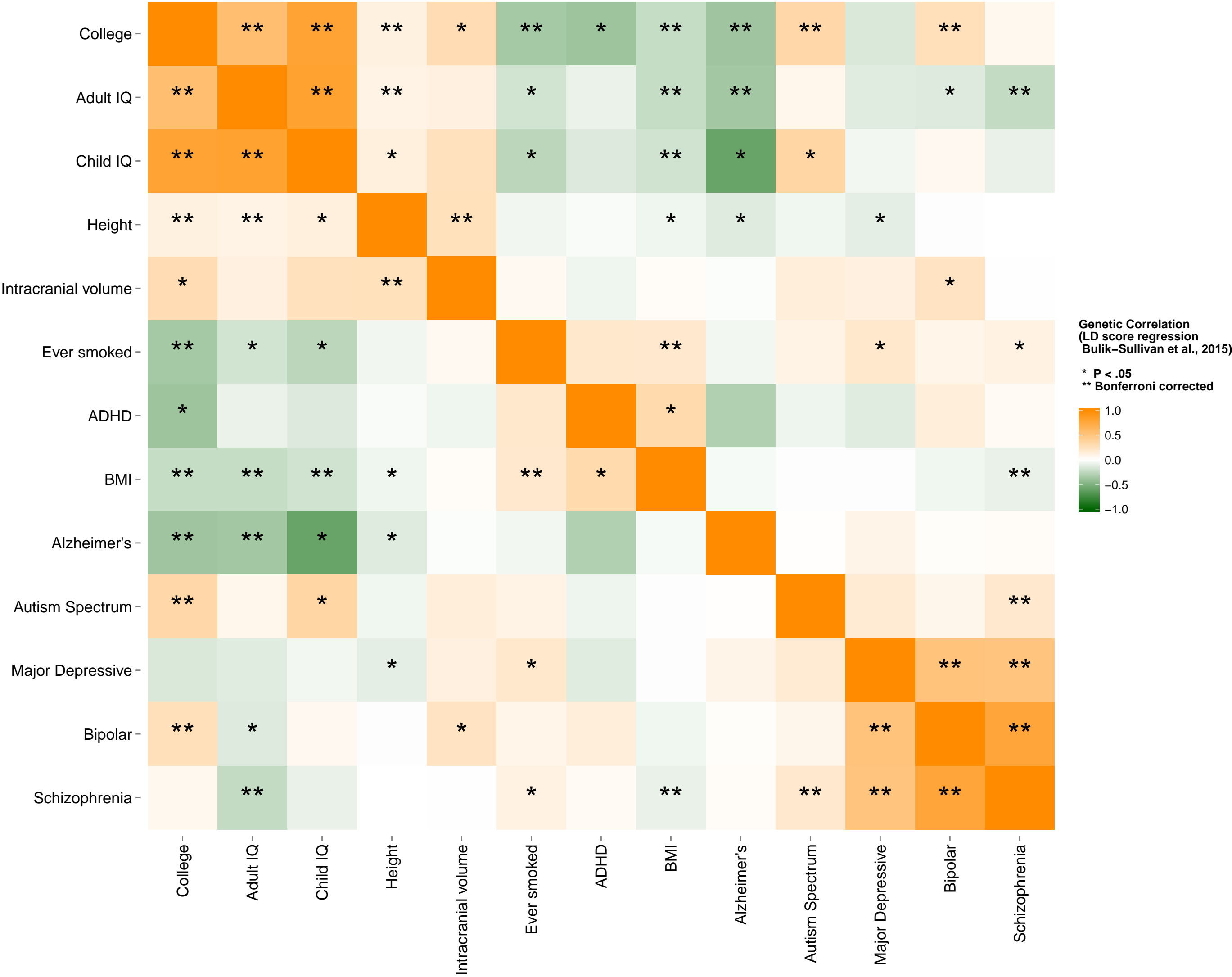

Supplement: Supplementary Figure 5 [file mp2015126x8.pdf]

# Correlations Genome-wide Polygenic Scores pT = 0.30

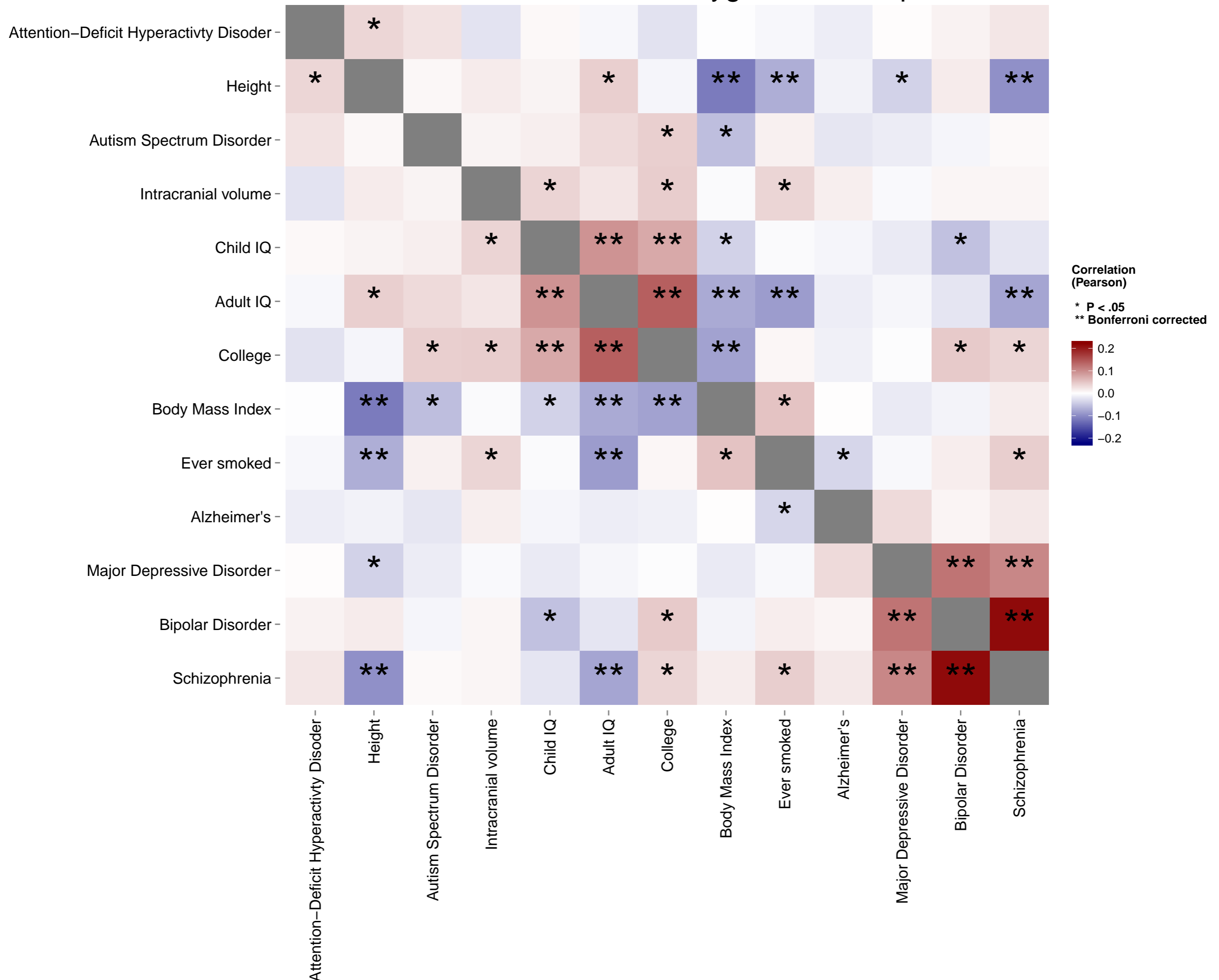

Supplement: Supplementary Figure 6a [file mp2015126x9.pdf]

# Correlations Genome-wide Polygenic Scores pT = 0.10

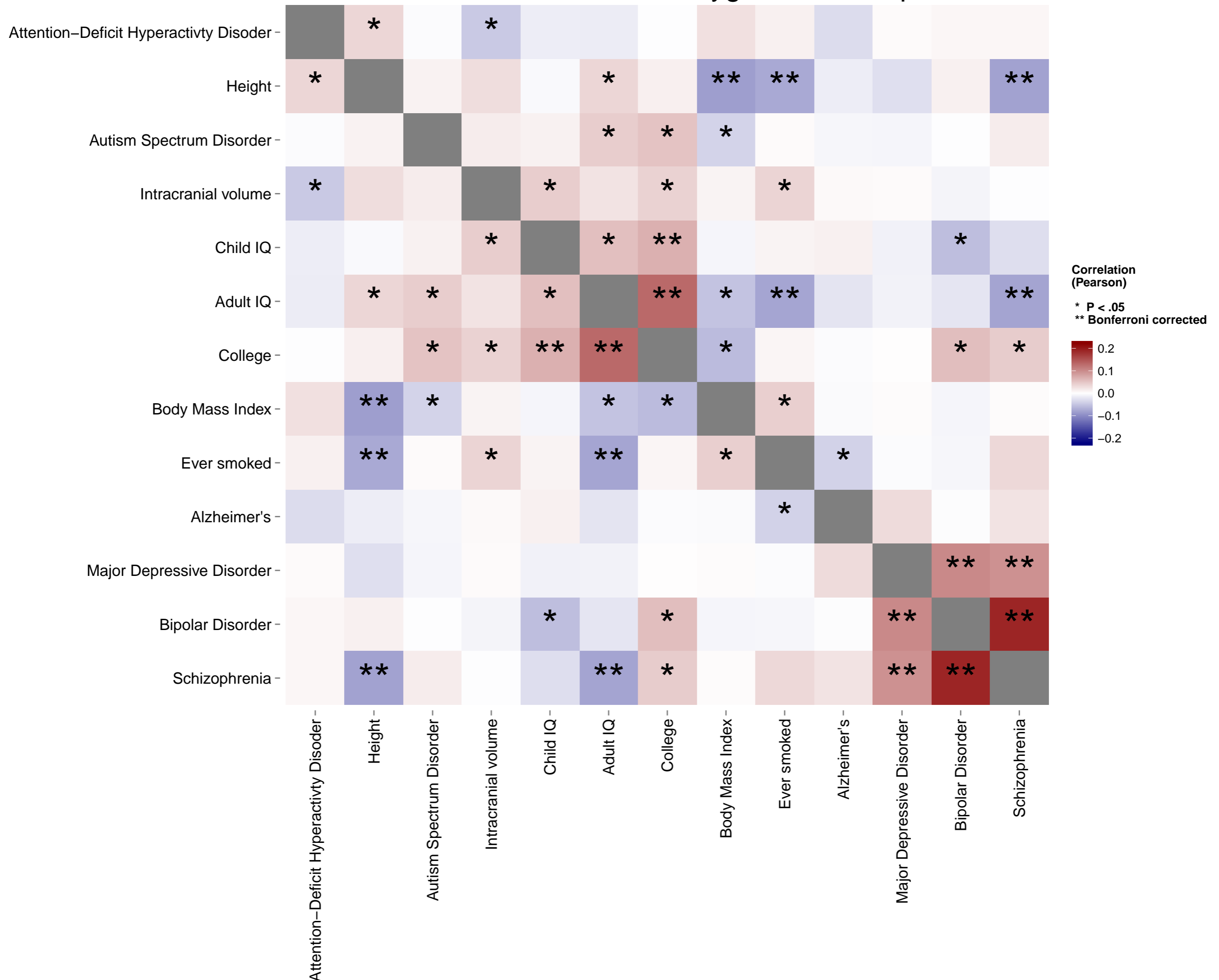

Supplement: Supplementary Figure 6b [file mp2015126x10.pdf]

# Correlations Genome-wide Polygenic Scores pT = 0.10

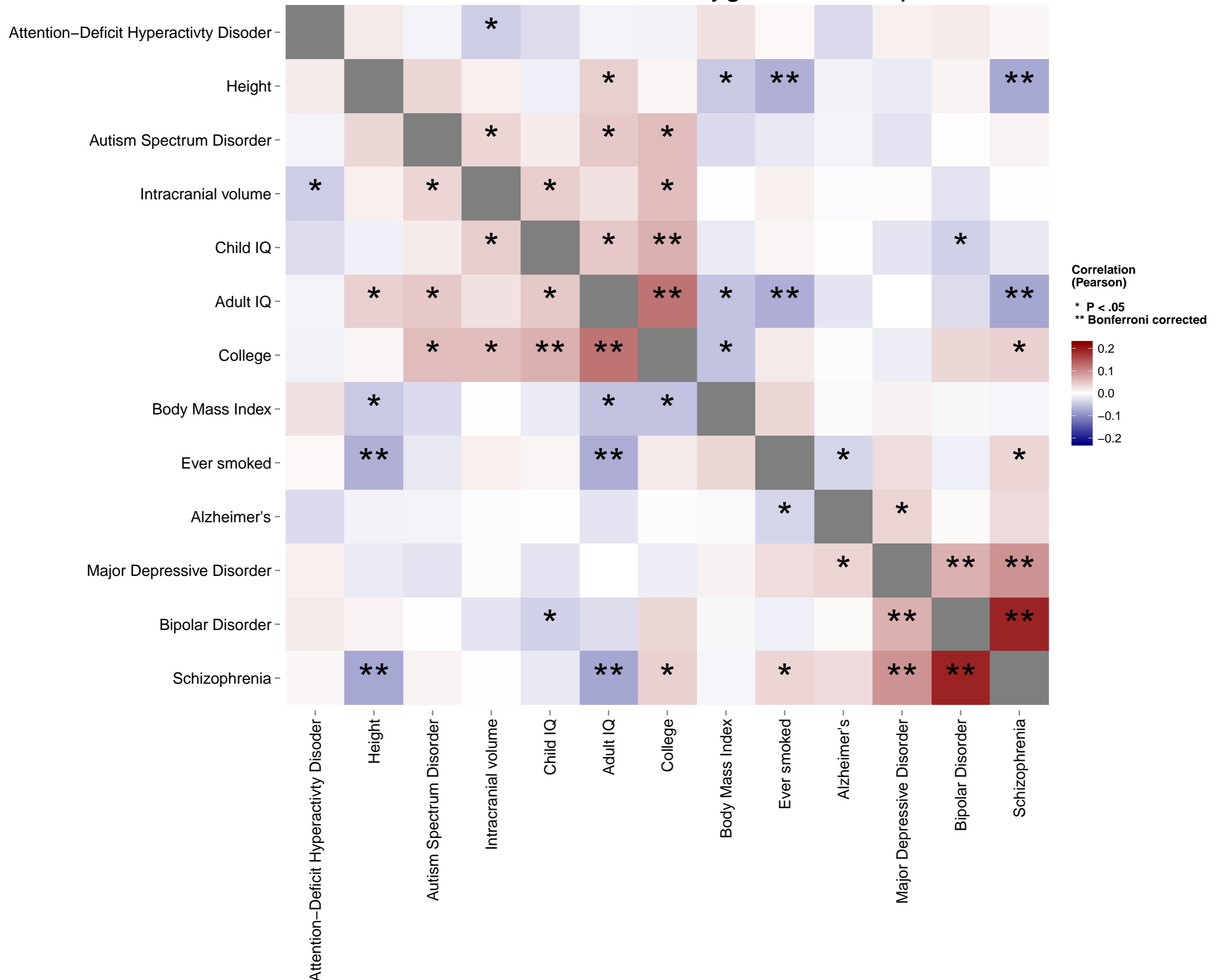

Supplement: Supplementary Figure 6c [file mp2015126x11.pdf]
