# Supplementary material for: Phenome-wide analysis of genome-wide polygenic scores
Source: Mol Psychiatry. 2015 Aug 25;21(9):1188–93. doi: 10.1038/mp.2015.126 (PMC4767701; doi:10.1038/mp.2015.126)
Supplement: Supplementary Figure 6 Legend [file mp2015126x12.pdf]

Supplementary Figure 6a-c. Correlations between 13 genome-wide polygenic scores. These results are based on GPS constructed using a GWAS P_T = 0.30, 0.10, and 0.05. *P* -values that pass Bonferroni correction are indicated with two asterisks, while those reaching nominal significance (thus suggestive evidence) are shown with a single asterisk.
