# Supplementary material for: Phenome-wide analysis of genome-wide polygenic scores
Source: Mol Psychiatry. 2015 Aug 25;21(9):1188–93. doi: 10.1038/mp.2015.126 (PMC4767701; doi:10.1038/mp.2015.126)
Supplement: Supplementary Methods 1 [file mp2015126x13.pdf]

Supplementary Methods 1 – Detailed Methods

This document contains supplementary material for: Krapohl et al. Phenome-wide analysis of genome-wide polygenic scores

MATERIALS AND METHODS

Sample

The sample was drawn from the Twins Early Development Study (TEDS), a multivariate longitudinal study that recruited over 16 000 twin pairs born in England and Wales in 1994, 1995 and 1996 [1,2]. TEDS has been shown to be representative of the UK population [3]. Supplementary Table 1 shows that the genotyped subsample of TEDS is representative of UK census data on key demographics from first contact through age 16 years. The project received approval from the Institute of Psychiatry ethics committee (05/Q0706/228) and parental consent was obtained before data collection.

Genotyping and quality control

SNP data were available for 3747 adolescents whose first language was English and who had no major medical or psychiatric problems. From that sample, 3665 DNA samples were successfully hybridized to Affymetrix GeneChip 6.0 SNP genotyping arrays (Affymetrix, Santa Clara, CA, USA) using standard experimental protocols as part of the WTCCC2 project (for details see Trzaskowski et al.) [4]. The nearly 700 000 genotyped SNPs were imputed to the 1000 Genomes reference panel (Phase I, v3, build 37 (hg19)) using IMPUTE v.2.3.0 software [5,6]. A total of 3152 DNA samples (1446 males and 1706 females) survived quality control criteria for ancestry, heterozygosity, relatedness and hybridization intensity outliers. For the present analyses we applied further quality control including: Minor allele frequencies > 0.01; Hardy-Weinberg equilibrium P<1x10-20; per-SNP missingness < 0.02; per-person missingness < 0.02. After quality control 4,285,205 SNPs remained that were entered into the polygenic score analyses. To control for ancestral stratification, we performed principal component analyses on a subset of 100,000 quality-controlled SNPs after removing SNPs in linkage disequilibrium ($r^2 > 0.2$) [7]. Using the Tracy–Widom test [8], we identified 8 axes with P < 0.05 that were used as covariates in the polygenic score analyses.

Summary Statistic Datasets

We obtained summary statistics from twelve GWAS [9–19] (Supplementary Table 2), which provided signed summary statistics and were imputed to at least HapMap2.

Summary-Summary Statistic Based Analysis

We estimate the genetic correlation between the base GWAS using a new technique by Bulik-Sullivan et al., [20] based on LD Score regression [21], which uses only GWAS summary statistics. This method exploits the fact that each effect-size estimate for a given SNP is a function of this SNP's linkage disequilibrium with other SNPs [21,22]. By quantifying extent to which the observed effect sizes can be explained by LD, the method estimates genetic effect on a trait, while controlling for confounding such as cryptic relatedness or population stratification. Similarly, the product of effect size statistics from two GWA studies of traits will index the covariance between these genetic effects. Normalizing the genetic covariance by the heritabilities estimates the genetic correlation between two traits.

Target phenotypes

Individuals were assessed on a wide range of phenotypes at the age of 16 including 50 traits from the domains of psychopathology, personality, cognitive abilities and educational achievement. A detailed description of all the phenotypic measures can be found in

Supplementary Methods 2. All measures were age- and sex-regressed and the z-score were used in the GPS analyses.

Polygenic score creation

We created polygenic scores from genome-wide SNP data of 3152 unrelated children using summary statistics from 12 published GWAS (Table 1). Strand-ambiguous SNPs were removed as well as the major histocompatibility complex region of the genome because of its complex linkage disequilibrium structure. Quality-controlled SNPs were pruned for linkage disequilibrium based on P-value informed clumping using r^2 = 0.1 cutoff within a 250-kb window to create a SNP-set in linkage equilibrium. The scores were calculated as the sum across SNPs of the number of reference alleles for each SNP multiplied by the effect size (β-coefficient) derived from the GWAS summary statistics. We employed two different methods of polygenic score creation:

First, we took the conventional approach and created genome-wide polygenic scores (GPS) that included variants exceeding three predefined P-value thresholds (P_T) in the base GWA summary statistics: 0.05, 0.10, 0.30.

Second, we performed high-resolution polygenic score prediction using the option provided by recently published PRSise software [23]. Specifically, for each individual, multiple polygenic scores were generated for all P-value thresholds (P_T) between P_T = 0.0001 and 0.50 at 0.0005 increments (i.e. at 999 thresholds) to identify the best-fit P-value threshold (P_T) for each pair of base and target phenotypes. PRSice defines 'best-fit' as the P_T at which the GPS predicts the target phenotype with the smallest p-value.

P-value thresholds and numbers of SNPs for the GPS for both methods are summarized in Supplementary Table 3-4. GPS were adjusted for 8 ancestry-informative dimensions (see above) for all analyses. Analyses were performed in R [24], PLINK [25,26], and PRSise software [23].

Multiple comparison correction

All phenotype-GPS association analyses, and the extremes analyses were performed in R, and the P-values obtained from each test were subsequently corrected for multiple testing using a "Nyholt-Šidák correction" based on the correlation matrix of variables; where the effective number of independent tests was calculated using the approach taken in Nyholt [27] and then used to compute a Šidák-corrected *P* value [28,29]. The basic idea of this approach is to "filter out" the correlations among the tests to arrive at the effective number of independent tests, which are then corrected for multiple testing. The multiple comparison adjustments were applied to an alpha of P = 0.05 for the conventional GPS analyses and extremes analyses; and of P = 0.001 for the high-resolution GPS analyses based on a simulation study by Euesden et al., [23]

Quantile analyses

We grouped individuals into GPS septiles and estimated the mean standardised phenotypic value as a function of GPS quantile. As for all other analyses, the model included covariates of sex, and age of data collection in the standardized mean phenotypic values; and ancestry-based PCs in the GPS quantiles.

References

1 Haworth CMA, Davis OSP, Plomin R. Twins Early Development Study (TEDS): A Genetically Sensitive Investigation of Cognitive and Behavioral Development From Childhood to Young Adulthood. *Twin Res Hum Genet* 2013; **16**: 117–125.

2 Oliver BR, Plomin R. Twins' Early Development Study (TEDS): A multivariate, longitudinal genetic investigation of language, cognition and behavior problems from childhood through adolescence. *Twin Res Hum Genet* 2007.

3 Kovas Y, Haworth CMA, Dale PS, Plomin R. The Genetic and Environmental Origins of Learning Abilities and Disabilities in the Early School Years. *Monogr Soc Res Child Dev* 2007; **72**: vii, 1–144.

4 Trzaskowski M, Eley TC, Davis OSP, Doherty SJ, Hanscombe KB, Meaburn EL *et al.* First Genome-Wide Association Study on Anxiety-Related Behaviours in Childhood. *PLoS ONE* 2013; **8**: e58676.

5 Howie B, Marchini J, Stephens M. Genotype Imputation with Thousands of Genomes. *G3 Genes Genomes Genet* 2011; **1**: 457–470.

6 Howie BN, Donnelly P, Marchini J. A Flexible and Accurate Genotype Imputation Method for the Next Generation of Genome-Wide Association Studies. *PLoS Genet* 2009; **5**: e1000529.

7 Fellay J, Shianna KV, Ge D, Colombo S, Ledergerber B, Weale M *et al.* A Whole-Genome Association Study of Major Determinants for Host Control of HIV-1. *Science* 2007; **317**: 944–947.

8 Patterson N, Price AL, Reich D. Population Structure and Eigenanalysis. *PLoS Genet* 2006; **2**: e190.

9 Rietveld CA, Medland SE, Derringer J, Yang J, Esko T, Martin NW *et al.* GWAS of 126,559 Individuals Identifies Genetic Variants Associated with Educational Attainment. *Science* 2013; **340**: 1467–1471.

10 Benyamin B, Pourcain Bs, Davis OS, Davies G, Hansell NK, Brion M-J *et al.* Childhood intelligence is heritable, highly polygenic and associated with FNBP1L. *Mol Psychiatry* 2014; **19**: 253–258.

11 Hibar DP, Stein JL, Renteria ME, Arias-Vasquez A, Desrivières S, Jahanshad N *et al.* Common genetic variants influence human subcortical brain structures. *Nature* 2015; **advance online publication**. doi:10.1038/nature14101.

12 Lambert JC, Ibrahim-Verbaas CA, Harold D, Naj AC, Sims R, Bellenguez C *et al.* Meta-analysis of 74,046 individuals identifies 11 new susceptibility loci for Alzheimer's disease. *Nat Genet* 2013; **45**: 1452–1458.

13 Psychiatric GWAS Consortium Bipolar Disorder Working Group. Large-scale genome-wide association analysis of bipolar disorder identifies a new susceptibility locus near ODZ4. *Nat Genet* 2011; **43**: 977–983.

14 Major Depressive Disorder Working Group of the Psychiatric GWAS Consortium, Ripke S, Wray NR, Lewis CM, Hamilton SP, Weissman MM *et al.* A mega-analysis of genome-wide association studies for major depressive disorder. *Mol Psychiatry* 2013; **18**: 497–511.

15 Schizophrenia Working Group of the Psychiatric Genomics Consortium. Biological insights from 108 schizophrenia-associated genetic loci. *Nature* 2014; **511**: 421–427.

16 Cross-Disorder Group of the Psychiatric Genomics Consortium. Identification of risk loci with shared effects on five major psychiatric disorders: a genome-wide analysis. *Lancet* 2013; **381**: 1371–1379.

17 Wood AR, Esko T, Yang J, Vedantam S, Pers TH, Gustafsson S *et al.* Defining the role of common variation in the genomic and biological architecture of adult human height. *Nat Genet* 2014; **46**: 1173–1186.

18 Tobacco and Genetics Consortium. Genome-wide meta-analyses identify multiple loci associated with smoking behavior. *Nat Genet* 2010; **42**: 441–447.

19 Locke AE, Kahali B, Berndt SI, Justice AE, Pers TH, Day FR *et al.* Genetic studies of body mass index yield new insights for obesity biology. *Nature* 2015; **518**: 197–206.

20 Bulik-Sullivan B, Finucane HK, Anttila V, Gusev A, Day FR, Consortium R *et al.* An Atlas of Genetic Correlations across Human Diseases and Traits. *bioRxiv* 2015; : 014498.

21 Bulik-Sullivan BK, Loh P-R, Finucane HK, Ripke S, Yang J, Schizophrenia Working Group of the Psychiatric Genomics Consortium *et al.* LD Score regression distinguishes confounding from polygenicity in genome-wide association studies. *Nat Genet* 2015; **47**: 291–295.

22 Yang J, Weedon MN, Purcell S, Lettre G, Estrada K, Willer CJ *et al.* Genomic inflation factors under polygenic inheritance. *Eur J Hum Genet EJHG* 2011; **19**: 807–812.

23 Euesden J, Lewis CM, O'Reilly PF. PRSice: Polygenic Risk Score software. *Bioinformatics* 2014; : btu848.

24 R Core Team. *R: A Language and Environment for Statistical Computing*. R Foundation for Statistical Computing: Vienna, Austria, 2014http://www.R-project.org/.

25 Chang CC, Chow CC, Tellier LC, Vattikuti S, Purcell SM, Lee JJ. Second-generation PLINK: rising to the challenge of larger and richer datasets. *GigaScience* 2015; **4**: 7.

26 Purcell S, Neale B, Todd-Brown K, Thomas L, Ferreira MAR, Bender D *et al.* PLINK: A Tool Set for Whole-Genome Association and Population-Based Linkage Analyses. *Am J Hum Genet* 2007; **81**: 559–575.

27 Nyholt DR. A Simple Correction for Multiple Testing for Single-Nucleotide Polymorphisms in Linkage Disequilibrium with Each Other. *Am J Hum Genet* 2004; **74**: 765–769.

28 Sidak Z. On Probabilities of Rectangles in Multivariate Student Distributions: Their Dependence on Correlations. *Ann Math Stat* 1971; **42**: 169–175.

29 O'Reilly PF, Hoggart CJ, Pomyen Y, Calboli FCF, Elliott P, Jarvelin M-R *et al.* MultiPhen: Joint Model of Multiple Phenotypes Can Increase Discovery in GWAS. *PLoS ONE* 2012; **7**: e34861.
