# Supplementary material for: Phenome-wide analysis of genome-wide polygenic scores
Source: Mol Psychiatry. 2015 Aug 25;21(9):1188–93. doi: 10.1038/mp.2015.126 (PMC4767701; doi:10.1038/mp.2015.126)
Supplement: Supplementary Methods 2 [file mp2015126x14.pdf]

Supplementary Information 2 – Description of phenotypic measures

This document contains supplementary material for: Krapohl et al. Phenome-wide analysis of genome-wide polygenic scores

Description of phenotypic measures:

‘g’: General cognitive ability. Mean of the standardized Ravens progressive matrixes [1] and the standardised Mill Hill Vocabulary test [2].

Ravens progressive matrixes [1]. This is a nonverbal ability task. This test consists of a series of incomplete patterns ("matrices"); in each case, the twin is asked to identify the missing part of the pattern. The twin does this by clicking on one of 8 possible missing parts.

Mill Hill Vocabulary test [2]. The test consists of a series of multiple-choice questions. For each item, a single word is presented at the top of the screen. Below it, six other words, labelled A, B, C, D, E and F are presented as the response options. Twins respond by clicking on the response they think is closest in meaning to the word at the top of the screen. There is only one correct response for each question.

General Certificate of Secondary Education (GCSE). The standardized UK-wide examination, the General Certificate of Secondary Education (GCSE), is taken by almost all (>99%) pupils at the end of compulsory education at typically at the age of 16 years. English, mathematics and science are compulsory subjects. The GCSE data for the present study were collected by questionnaires sent by mail and by telephone interview of parents and twins themselves. After completed forms were received from the families, the grades were coded from 11 (the highest grade: A*) to 4 (the lowest pass grade: G); no information about failed results was available. For 1729 individuals, self- and parent-reported GCSE results were verified using data obtained from the UK National Pupil Database,[3] yielding correlations of 0.99 for mathematics, 0.98 for English and 0.96 for science. The GCSE measures used in the present analyses were the three compulsory core subjects: mathematics (highest recorded grade for mathematics qualification at GCSE), English (highest grade recorded or ‘English Language’ and ‘English Literature’), and science (highest grade of any science subjects taken).

PISA attitudes to School (OECD Programme for International Student Assessment): www.pisa.oecd.org. TEDS measures were selected from the PISA 2000, 2003 and 2006 student questionnaires. This scale measured the attitudes the twins have about school and how it prepares them for life after school. Participants were asked to rate how strongly they agree with statements on a 4-point scale. For example: School has done little to prepare me for adult life when I leave school; School has helped give me confidence to make decisions.

PISA Homework total. Mean of the items from the PISA Homework Behavior and PISA Homework Feedback.

PISA homework behaviour scale. (OECD Programme for International Student Assessment): www.pisa.oecd.org. TEDS measures were selected from the PISA 2000, 2003 and 2006 student questionnaires. This scale assessed how children do their homework. Children were asked to indicate which statement applies to them on a 4-point scale. For example: I complete my homework on time; I do my homework while watching television.

PISA homework feedback scale (OECD Programme for International Student Assessment): www.pisa.oecd.org. TEDS measures were selected from the PISA 2000, 2003 and 2006 student questionnaires. This scale measured the feedback participants got for the homework handed in. Children were asked to indicate which statement applies to them on a 4-point scale. For example: My teachers make useful comments on my homework.

PISA math self-efficacy: child-reported questionnaire, selected from the PISA 2000, 2003 and 2006 student questionnaires (OECD Programme for International Student Assessment): www.pisa.oecd.org. 8 items. For example: How confident do you feel about having to do the following mathematics tasks? Solving an equation like 3x + 5 = 17

PISA math interest**:** child-reported questionnaire, selected from the PISA 2000, 2003 and 2006 student questionnaires (OECD Programme for International Student Assessment): www.pisa.oecd.org. 3 items. For example: I do mathematics because I enjoy it.

PISA time spent on math: child-reported questionnaire, selected from the PISA 2000, 2003 and 2006 student questionnaires (OECD Programme for International Student Assessment): www.pisa.oecd.org. 3 items. For example: Thinking about the past year. How much time did you typically spend per week studying mathematics? Study or homework in mathematics by myself?

Academic self-concept [4]. This is a child-reported 11-item questionnaire shortened from a 20-item 5-point scale from 'Very much like me' to 'Not like me at all'. For example: I like having difficult work to do; I'm clever.

GRIT. Mean of GRIT, Perseverance of interest and GRIT consistency of interest.

- GRIT, Perseverance of interest [5] Twins were asked: To what extent do the following statements describe you? Participants were asked to rate the statements on a 5-point scale from very much like me to not like me at all. For example: I have difficulty maintaining my focus on projects that take more than a few months to complete; Setbacks don't discourage me.
- GRIT, Consistency of interest [5] Twins were asked: To what extent do the following statements describe you? Participants were asked to rate the statements on a 5-point scale from very much like me to not like me at all. For example: New ideas distract me; Set a goal; maintain focus.

Personality Big Five-factor model [6] Twins were told that these questions are about personality and were asked to report where they fall on the scale from high to low on a 5-point scale.
Neuroticism. For example: rating anxiousness from fearful, apprehensive to relaxed, unconcerned.
Extraversion. For example: rating 'assertiveness' from dominant, forceful to unassuming, quiet, resigned.
Openness. For example: rating 'ideas' from strange, odd, peculiar, creative to pragmatic, rigid
Agreeableness. For example: rating 'compliance' from docile, cooperative to oppositional, combative, aggressive.
Contentiousness. For example: rating 'deliberation' from cautious, ruminative, reflective to hasty, careless, rash.

Life Satisfaction [7]. Multidimensional students' life satisfaction scale. Participants were asked to think about the past few months and rate how much they agree with the statements presented on a 6-point scale ranging from strongly agree to strongly disagree. This measure is the mean of five subscales:

- Life satisfaction Family. For example: My family is better than most; I enjoy being at home with my family.
- Life satisfaction Friends. For example: I have a bad time with my friends; My friends will help me if I need it.
- Life satisfaction School. For example: I learn a lot at school; There are many things about school I don't like.
- Life satisfaction Living Environment. For example: There are lots of fun things to do where I live; I wish there were different people in my neighbourhood/area.
- Life satisfaction Self. For example: I am fun to be around; There are lots of things I can do well.

Curiosity Explore [8]. Twins were asked: To what extent do you agree with the following statements? The participants had to rate the statements on a 7-point scale from strongly agree to strongly disagree. For example: I would describe myself as someone who actively

seeks as much information as I can in a new situation; I frequently find myself looking for new opportunities to grow as a person (e.g., information, people, resources).

Curiosity Flow [8]. Twins were asked: To what extent do you agree with the following statements? The participants rated the statements on a 7-point scale from strongly agree to strongly disagree. For example: When I am participating in an activity, I tend to get so involved that I lose track of time; When I am actively interested in something, it takes a great deal to interrupt me.

CHAOS (Confusion, Hubbub and Order Scale) at home [9]. This is a home environment scale, measuring the household disorganization. Data were collected from twins, who rated statements on a 3-point scale from not true to very true. For example: It's a real zoo" in our home; you can't hear yourself think in our home.

Attachment total. Inventory of Parent and Peer Attachment. Just the peer attachment was included, which represents the mean of 3 subscales, adapted from Armsden & Greenberg [10]. In all cases twins rated the statements on 5-point scale:

- Attachment trust scale. For example: I can count on my friends when I need to get something off my chest.
- Attachment communication scale. For example; My friends encourage me to talk about my difficulties.
- Attachment alienation scale. For example: It seems as if my friends are irritated with me for no reason.

Parental control. This is a measure of home environment and relationships. Adapted from the National Institute of Child Health and Development (NICHD) Early Child Care and Youth Development (SECCYD) study. Parental control measures how decisions about twins' daily lives are reached and how much control parents exercise about them. For example: How do you make most of the decisions about the following topics? How late can you stay up on school night? Which friends you can spend time with etc. Twins rated these items as follows: My parent(s) decide, my parents decide after discussing with me, we decide together, I decide after discussing it with my parents, I decide all by myself.

Parental monitoring. This is a measure of home environment and relationships. Adapted from NICHD early childcare and youth development study. The parental monitoring scale, rated by the twins , assesses how much a parent knows about the lives of the children and what they do. For example, the twins rated the following questions: How much does a parent or an adult in your home know about: Where you go right after school? Who you spend your time with? Etc.

Peer victimization. Shortened 6-item version of the Child-reported Multidimensional Peer-Victimization Scale [11]. Contains four main factors —physical victimization, verbal victimization, social manipulation, and attacks on property.

SDQ Hyperactivity scale [12] A parent-reported dimensional and developmental measure of child mental health. Five items assess three key symptom domains of attention-deficit/hyperactivity disorder (ADHD): inattention (2 items), hyperactivity (2 items), and impulsiveness (1 item), using a 3-point Likert scale (Not true; Quite true; Very true). Example: My child is restless, overactive, and cannot stay still for long.
SDQ Conduct scale [12]. This is a parent-reported dimensional and developmental measure of child mental health. Five items measuring externalising behaviour problems on 3-point Likert scale (Not true; Quite true; Very true). For example: Often fights with other children or bullies them; Often has temper tantrums or hot temper.
SDQ Prosocial scale [12]. This is a parent-reported dimensional and developmental measure of child mental health. Five items measure prosocial behaviour on 3-point Likert scale (Not true; Quite true; Very true). For example: Considerate of other people's feelings; Helpful if someone is hurt, upset or feeling ill; Often volunteers to help others (parents, teachers, other children).
Autism Quotient Social scale: [13]. This is a parent-reported subscale of the Autism Quotient (AQ), a measure of autistic-like traits in the general population. 9 items measuring social-

communication deficits on 4-point Likert scale (definitely agree; slightly agree slightly disagree; definitely disagree). For example: Finds social situations easy; Finds it hard to make new friends.

Autism quotient Attention Switching scale [13]. This is a parent-reported subscale of the Autism Quotient (AQ), a measure of autistic-like traits in the general population. 8 items measuring social-communication deficits on 4-point Likert scale (definitely agree; slightly agree slightly disagree; definitely disagree). For example: Does not get upset if his/her daily routine is disturbed; Enjoys doing things spontaneously.

Autism Quotient Imagination [13]. This is a parent-reported subscale of the Autism Quotient (AQ), a measure of autistic-like traits in the general population. 6 items measuring social-communication deficits on 4-point Likert scale (definitely agree; slightly agree slightly disagree; definitely disagree). For example: If trying to imagine something, finds it easy to create a picture in his/her mind; When reading a book, can easily imagine what the characters might look like.

Autism Quotient Attention to Detailscale [13]. This is a parent-reported subscale of the Autism Quotient (AQ), a measure of autistic-like traits in the general population. 5 items measuring attention to detail on 4-point Likert scale (definitely agree; slightly agree slightly disagree; definitely disagree). For example: Usually notices car number plates or similar strings of information; Notices patterns in things all the time.

ARBQ Anxiety scale [14]. This is a parent-reported, 19-item questionnaire on anxiety-related behaviors in children. The items (three-point scale: never, sometimes, often) represent five dimensions: General Distress (negative mood) (for example: Often seems worked up, on edge or tense ; Separation Anxiety (for example: Is often extremely upset or distressed when parent leaves, wound up or stressed ; Fears (for example: Is afraid of animals or insects (like dogs, spiders, or snakes); Obsessive-Compulsive Behaviors (for example: Tends to check things are done exactly right), and Shyness/Inhibition (for example: Tends to be shy and timid).

Conners Impulsivity scale (CPRS-R) [15]. This is a parent-reported subscale of the Revised Conners' Parent Rating Scale, which consists of 9 items (4-point Likert scale) measuring impulsive behaviors. For example: Interrupts conversations.

Conners Inattention scale. This is a parent-reported subscale of the Revised Conners' Parent Rating Scale (CPRS-R) [15], which includes 9 items (4-point Likert scale) measuring inattention problems. For example: Does not follow through on instructions and fails to finish work, schoolwork or chores.

Callous Unemotional Traits. Parent-reported Inventory of Callous-Unemotional Traits (ICU) [16,17]; 24-item questionnaire designed to provide a comprehensive assessment of callous and unemotional traits. These traits have proven to be important for designating a distinct subgroup group of antisocial and aggressive youth. The ICU has three subscales: Callousness, Uncaring, and Unemotional.

SANS (negative symptoms). The parent-reported Scale for the Assessment of Negative Symptoms (SANS) [18] measures negative symptoms in schizophrenia. SANS is the mean of 5 domains, and within each domain separate symptoms are rated from 0 (absent) to 5 (severe).

- Affective Flattening or Blunting (Unchanging Facial Expression; Decreased Spontaneous Movements; Paucity of Expressive Gestures; Poor Eye Contact; Affective Nonresponsivity; Lack of Vocal Inflections; Global Rating of Affective Flattening; Inappropriate Affect)
- Alogia (Poverty of Speech; Poverty of Content of Speech; Blocking; Increased Latency of Response; Global Rating of Alogia)
- Avolition - Apathy (Grooming and Hygiene; Impressistence at Work or School; Physical Anergia; Global Rating of Avolition – Apathy)
- Anhedonia - Asociality (Recreational Interests and Activities; Sexual Interest and Activity; Ability to Feel Intimacy and Closeness; Relationships with Friends and Peers; Global Rating of Anhedonia-Asociality)

- Attention (Social Inattentiveness; Inattentiveness During Mental Status Testing; Global Rating of Attention; Scale for the Assessment of Positive Symptoms (SAPS))

Paranoid consists of self-report ratings on a 15-item questionnaire [19] from the Paranoia Checklist [20], which assesses paranoid symptomatology in a non-clinical population.

Cognitive disorganization includes 11 items from the short Oxford-Liverpool Inventory of Feelings and Experiences [21], which assesses shizoptyic symptoms in the population.

Grandiosity was assessed with items from the "Myself" subscale of Cognition Checklist for Mania-Revised [22], the Peters and colleagues' Delusions Inventory [23], and items developed from clinical case studies.

BMI: Body Mass Index (BMI), derived from self-reported height and weight. BMI = $\frac{\text{weight}(kg)}{(\text{height}(m))\text{x}^2}$

Height: self-reported height.

Puberty [24]. This is a child-reported Pubertal Development Scale, with 9 items measuring pubertal state for both sexes. For example: Would you say that your body hair (underarm & pubic) growth has [Not yet begun/Barely begun/Definitely begun/Completed/Not sure].

Insomnia [25]. This is a child-reported Insomnia Severity Scale, 2ith 7 items (5-point scale) measuring perceived sleep difficulties. Examples: Difficulty falling asleep; Problems staying asleep.

Sleep total [26]: child-reported Pittsburgh Sleep Quality Index. 9 items assessing sleep quality. Example: "During the past month, how often have you had trouble sleeping because you: Had bad dreams".

References

1 Raven J, Court J, Raven J. *Manual for Raven's Progressive Matrices and Vocabulary Scales*. Oxford University Press: Oxford, 1996.

2 Raven J, Raven J, Court J. *Mill Hill vocabulary scale*. OPP: Oxford, 1998.

3 National pupil database - GOV.UK. https://www.gov.uk/government/collections/national-pupil-database (accessed 10 Sep2014).

4 Burden R. Assessing Children's Perceptions of Themselves as Learners and Problem-Solvers The Construction of the Myself-as-Learner Scale (MALS). *Sch Psychol Int* 1998; **19**: 291–305.

5 Duckworth AL, Quinn PD. Development and Validation of the Short Grit Scale (Grit–S). *J Pers Assess* 2009; **91**: 166–174.

6 Mullins-Sweatt SN, Jamerson JE, Samuel DB, Olson DR, Widiger TA. Psychometric Properties of an Abbreviated Instrument of the Five-Factor Model. *Assessment* 2006; **13**: 119–137.

7 Huebner ES. Preliminary development and validation of a multidimensional life satisfaction scale for children. *Psychol Assess* 1994; **6**: 149.

8 Kashdan TB, Rose P, Fincham FD. Curiosity and Exploration: Facilitating Positive Subjective Experiences and Personal Growth Opportunities. *J Pers Assess* 2004; **82**: 291–305.

9 Matheny AP, Wachs TD, Ludwig JL, Phillips K. Bringing order out of chaos: Psychometric characteristics of the confusion, hubbub, and order scale. *J Appl Dev Psychol* 1995; **16**: 429–444.

10 Armsden GC, Greenberg MT. The inventory of parent and peer attachment: Individual differences and their relationship to psychological well-being in adolescence. *J Youth Adolesc* 1987; **16**: 427–454.

11 Mynard H, Joseph S. Development of the multidimensional peer-victimization scale. *Aggress Behav* 2000; **26**: 169–178.

12 Goodman R. The Strengths and Difficulties Questionnaire: A Research Note. *J Child Psychol Psychiatry* 1997; **38**: 581–586.

13 Baron-Cohen S, Wheelwright S, Skinner R, Martin J, Clubley E. The autism-spectrum quotient (AQ): evidence from Asperger syndrome/high-functioning autism, males and females, scientists and mathematicians. *J Autism Dev Disord* 2001; **31**: 5–17.

14 Eley TC, Bolton D, O'Connor TG, Perrin S, Smith P, Plomin R. A twin study of anxiety-related behaviours in pre-school children. *J Child Psychol Psychiatry* 2003; **44**: 945–960.

15 Conners CK, Sitarenios G, Parker JDA, Epstein JN. The Revised Conners' Parent Rating Scale (CPRS-R): Factor Structure, Reliability, and Criterion Validity. *J Abnorm Child Psychol* 1998; **26**: 257–268.

16 Kimonis ER, Frick PJ, Skeem JL, Marsee MA, Cruise K, Munoz LC *et al.* Assessing callous–unemotional traits in adolescent offenders: Validation of the Inventory of Callous–Unemotional Traits. *Int J Law Psychiatry* 2008; **31**: 241–252.

17 Frick PJ. The inventory of callous-unemotional traits. *Unpubl Rat Scale* 2004.

18 Andreasen NC. Scale for the assessment of negative symptoms. *Iowa City Univ Iowa* 1984.

19 Ronald A, Sieradzka D, Cardno AG, Haworth CMA, McGuire P, Freeman D. Characterization of Psychotic Experiences in Adolescence Using the Specific Psychotic Experiences Questionnaire: Findings From a Study of 5000 16-Year-Old Twins. *Schizophr Bull* 2014; **40**: 868–877.

20 Freeman D, Garety PA, Bebbington PE, Smith B, Rollinson R, Fowler D *et al.* Psychological investigation of the structure of paranoia in a non-clinical population. *Br J Psychiatry* 2005; **186**: 427–435.

21 Mason O, Linney Y, Claridge G. Short scales for measuring schizotypy. *Schizophr Res* 2005; **78**: 293–296.

22 Beck AT, Colis MJ, Steer RA, Madrak L, Goldberg JF. Cognition Checklist for Mania—Revised. *Psychiatry Res* 2006; **145**: 233–240.

23 Peters E, Joseph S, Day S, Garety P. Measuring Delusional Ideation: The 21-Item Peters et al. Delusions Inventory (PDI). *Schizophr Bull* 2004; **30**: 1005–1022.

24 Petersen AC, Crockett L, Richards M, Boxer A. A self-report measure of pubertal status: Reliability, validity, and initial norms. *J Youth Adolesc* 1988; **17**: 117–133.

25 Bastien CH, Vallières A, Morin CM. Validation of the Insomnia Severity Index as an outcome measure for insomnia research. *Sleep Med* 2001; **2**: 297–307.

26 Buysse DJ, Reynolds III CF, Monk TH, Berman SR, Kupfer DJ. The Pittsburgh sleep quality index: A new instrument for psychiatric practice and research. *Psychiatry Res* 1989; **28**: 193–213.
