# Supplementary material for: Phenome-wide analysis of genome-wide polygenic scores
Source: Mol Psychiatry. 2015 Aug 25;21(9):1188–93. doi: 10.1038/mp.2015.126 (PMC4767701; doi:10.1038/mp.2015.126)
Supplement: Supplementary Methods 3 [file mp2015126x15.pdf]

Supplementary Methods 3 – Illustration: Power calculations

This document contains supplementary material for: Krapohl et al. Phenome-wide analysis of genome-wide polygenic scores

A genome-wide polygenic score (GPS) explaining 0.5% of the variance (i.e., 1% of the heritable variance for a trait that is 50% heritable) with 80% power could, for example, arise under the following circumstances (calculations based on equations published by Dudbridge et al.,[1]) :

- 63,308: number of independent SNPs in the polygenic score
- Range of p-value for selection from training sample: 0.00 – 0.30
- Proportion of SNPs with no effects on training trait: 0.95
- Discovery and target sample sizes: of 71,000 and 1,567, respectively
- Binary trait in discovery sample; continuous trait in target sample
- Disease prevalence in both samples: 1%
- Sampling fraction: 0.75 in discovery sample and 0.01 (population prevalence) in target sample
- Total variance that is explained by genetic effects in discovery sample: 18%
- Covariance between genetic effect sizes in the two populations: 6% (e.g. genetic effects on trait 1 = 18%; genetic effects on trait 2 = 18%; genetic correlation = 0.33)"

References

1 Dudbridge F. Power and Predictive Accuracy of Polygenic Risk Scores. *PLoS Genet* 2013; **9**: e1003348.
