# Supplementary material for: Phenome-wide analysis of genome-wide polygenic scores
Source: Mol Psychiatry. 2015 Aug 25;21(9):1188–93. doi: 10.1038/mp.2015.126 (PMC4767701; doi:10.1038/mp.2015.126)
Supplement: Supplementary Table 1 [file mp2015126x16.pdf]

	Returned data (N families)	% parents A-levels or higher	% Mother employed	% Father employed	% Female
UK census[a]	--	32%	49%	89%	50%
TEDS 1st contact genotyped subsample	3,152	29%	47%	89%	54%
TEDS age 16 genotyped subsample	2,886	31%	47%	89%	55%

a The UK 2001 census data (ONS, 2001) for families with children were used because they represent more appropriate comparisons than UK 2011 census data for TEDS twins who were born 1994 -1996.
