# Supplementary material for: Phenome-wide analysis of genome-wide polygenic scores
Source: Mol Psychiatry. 2015 Aug 25;21(9):1188–93. doi: 10.1038/mp.2015.126 (PMC4767701; doi:10.1038/mp.2015.126)
Supplement: Supplementary Table 2 [file mp2015126x17.pdf]

Trait	binary/continuous	Sample size	Reference
College	binary	120,917	Rietveld, et al., Science, 2013
Adult IQ	continuous	53,949	Davies, et al., Mol Psych, 2015
Child IQ	continuous	9,616*	Benyamin, et al., Mol Psych, 2014
Intracranial Volume	continuous	11,373	Hibar, et al., Nature, 2015
Alzheimer's disease	binary	54,162	Lambert, et al., Nat Genet, 2013
Bipolar disorder	binary	16,731	PGC Bipolar Working Group, Nat Genet, 2011
Major Depressive Disorder	binary	18,759	PGC MDD Working Group, Mol Psych, 2013
Schizophrenia	binary	70,100	PGC Schizophrenia Working Group, Nature, 2014
Attention-Deficit Hyperactivty Disorder	binary	5,422	PGC Cross-Disorder Group, Lancet, 2013
Autism Spectrum Disorder	binary	10,263	PGC Cross-Disorder Group, Lancet, 2013
Body Mass Index	continuous	322,154	Locke, et al., Nat Genet, 2015
Height	continuous	253,280	Wood, et al., Nat Gen, 2014
Ever/Never Smoked	binary	74,035	TAG Consortium, Nat Genet, 2010
