# Supplementary material for: Phenome-wide analysis of genome-wide polygenic scores
Source: Mol Psychiatry. 2015 Aug 25;21(9):1188–93. doi: 10.1038/mp.2015.126 (PMC4767701; doi:10.1038/mp.2015.126)
Supplement: Supplementary Information [file mp2015126x20.docx]

**Supplementary Information:**

**Supplementary Methods 1.** Detailed Methods

**Supplementary Methods 2.** Description phenotypic measures

**Supplementary Methods 3.** Exemplary Power calculation

**Supplementary Table 1.** The genotyped subsample of the Twin Early Development Study (TEDS) is representative of UK census data from first contact through age 16.

**Supplementary Table 2.** Sample sizes and references for GWAS summary statistics used to create GPS. *The child IQ GWAS summary statistics used in the present study are based on a re-analysis of the data, which excluded the Twins Early Development Study used as the target sample in the present analyses.

**Supplementary Table 3.** Detailed statistics for correlations between 13 genome-wide polygenic scores and 50 traits from the behavioral phenome. These results are based on GPS constructed using a GWAS *P*_T_ = 0.30, 0.10 and 0.05.

**Supplementary Table 4.** Detailed statistics for correlations between 13 genome-wide polygenic scores and 50 traits from the behavioral phenome. These results are based on GPS constructed using a GWAS *P*_T_ =Best-Fit, i.e. a high-resolution polygenic scoring approach, implemented in the software PRSice^1^, that identifies the most predictive GPS for each phenotype.

**Supplementary Figure 1.** Correlations between 13 genome-wide polygenic scores and 50 traits from the behavioral phenome. These results are based on GPS constructed using a GWAS *P*_T_ =0.10 and 0.05. *P*-values that pass Nyholt-Sidak correction (see Supplementary Method 1) are indicated with two asterisks, while those reaching nominal significance (thus suggestive evidence) are shown with a single asterisk.

**Supplementary Figure 2.** Correlations between 13 genome-wide polygenic scores and 50 traits from the behavioral phenome. These results are based on GPS constructed using a GWAS *P*_T_ = Best-Fit, i.e. a high-resolution polygenic scoring approach, implemented in the software PRSice^1^, that identifies the most predictive GPS for each phenotype. *P*-values that pass Nyholt-Sidak correction (see Supplementary Method section) are indicated with two asterisks, while those reaching nominal significance at *P* < 0.001 (a nominal significance threshold suggested by simulations published in Euesden et al., 2015) are shown with a single asterisk.

**Supplementary Figure 3a-c.** Distributions of 12 genome-wide polygenic scores (GPS). The normal distribution is imposed on top of the histogram for individual GPS scores. The GPS are re-scaled, so that, the X-axis refers to standard deviation units and the mean is zero. The colored tails of the distribution indicate 1% and 5% cut-offs. The GPS are scored in the direction implied by the name of the trait; for example, a high score on the Alzheimer’s GPS indicates high genetic risk for Alzheimer’s. Similarly normal distributions emerged for *P*-value thresholds of 0.30, 0.10 and 0.05, although the distributions in this figure using the 0.30 threshold are smoother because more SNPs were selected for these GPS.

**Supplementary Figure 4.** Phenotypic correlations (Pearson’s *r* ) between the 50 phenotypic traits assessed at age 16. See detailed statistics in Supplementary Table 3 and detailed description of the measures in Supplementary Methods 2.

**Supplementary Figure 5.** Genetic correlations between the summary statistics of discovery GWAS that were subsequently used to create GPS. Correlations were estimated using bivariate LD score regression (Bulik-Sullivan et al., 2015). The direction of the correlations is indicated by the coloring (orange for positive, green for negative) and the magnitude of the correlations is indicated by the color gradient. Single asterisks indicate nominal P-value <0.05. Correlations that are significantly different from zero after Bonferroni correction have two asterisks.

Supplementary information is available at the Molecular Psychiatry website.
